# Combined analysis of RNA‐sequence and microarray data reveals effective metabolism‐based prognostic signature for neuroblastoma

**DOI:** 10.1111/jcmm.15650

**Published:** 2020-07-19

**Authors:** Xinyao Meng, Chenzhao Feng, Erhu Fang, Jiexiong Feng, Xiang Zhao

**Affiliations:** ^1^ Department of Pediatric Surgery Tongji Hospital Tongji Medical College Huazhong University of Science and Technology Wuhan P. R. China; ^2^ School of Basic Medicine Tongji Medical College Huazhong University of Science and Technology Wuhan P. R. China

**Keywords:** long non‐coding RNA, metabolism, neuroblastoma, prognosis, signature, stage 4s

## Abstract

The relationship between metabolism reprogramming and neuroblastoma (NB) is largely unknown. In this study, one RNA‐sequence data set (n = 153) was used as discovery cohort and two microarray data sets (n = 498 and n = 223) were used as validation cohorts. Differentially expressed metabolic genes were identified by comparing stage 4s and stage 4 NBs. Twelve metabolic genes were selected by LASSO regression analysis and integrated into the prognostic signature. The metabolic gene signature successfully stratifies NB patients into two risk groups and performs well in predicting survival of NB patients. The prognostic value of the metabolic gene signature is also independent with other clinical risk factors. Nine metabolism‐related long non‐coding RNAs (lncRNAs) were also identified and integrated into the metabolism‐related lncRNA signature. The lncRNA signature also performs well in predicting survival of NB patients. These results suggest that the metabolic signatures have the potential to be used for risk stratification of NB. Gene set enrichment analysis (GSEA) reveals that multiple metabolic processes (including oxidative phosphorylation and tricarboxylic acid cycle, both of which are emerging targets for cancer therapy) are enriched in the high‐risk NB group, and no metabolic process is enriched in the low‐risk NB group. This result indicates that metabolism reprogramming is associated with the progression of NB and targeting certain metabolic pathways might be a promising therapy for NB.

## 1 INTRODUCTION

Neuroblastoma (NB) is a childhood tumour originating from sympathetic nervous system.^1^ It is the most common extracranial solid tumour of childhood, affecting about 1 in 7000 live births.^1^, ^2^, ^3^ According to the Children's Oncology Group (COG), NB patients are stratified into low‐, intermediate‐ and high‐risk groups.^3^ This risk classification is based on age at diagnosis, MYCN amplification status, International Neuroblastoma Staging System (INSS) stage, histopathology and tumour cell ploidy.^3^, ^4^ Patients with high‐risk NB still demonstrate poor survival outcome (<50%) despite multimodal aggressive therapy.^4^ While NB patients with metastatic stage 4 disease are at high risk for death from refractory disease, patients with metastatic stage 4s disease generally had a surprisingly good prognosis and even underwent spontaneous regression without tumour‐specific therapy.^4^, ^5^, ^6^, ^7^ The spontaneous regression of NB has been validated by mass screening programmes,^8^, ^9^, ^10^, ^11^ and it is most evident in patients with stage 4s disease.^12^, ^13^, ^14^, ^15^ Patients with stage 4s NB generally had a small primary tumour but with metastatic disease in the liver, skin and bone marrow, or any combination of these.^12^ In fact, spontaneous regression is not restricted to stage 4s NB and can be seen in patients with any stage of NB.^5^ Since spontaneous regression of NB is most evident in infants with stage 4s disease, investigators have focused on stage 4s tumours as a surrogate to investigate the mechanisms that underlie spontaneous regression.^13^, ^14^, ^15^ However, the underlying mechanism responsible for the spontaneous regression of NB is still largely unknown.

In recent years, it is becoming evident that changes in cell metabolism contribute to cancer development and progression.^16^, ^17^, ^18^, ^19^, ^20^ Converging evidence indicates that the well‐known tumour suppressor p53 controls multiple metabolic pathways and plays a major role in metabolism of cancer cells.^20^ Researchers have also found that MYCN oncogene is involved in metabolic processes: MYCN could enhance glutaminolysis and induce oxidative stress by producing reactive oxygen species (ROS),^21^ and could also negatively regulate monocarboxylate transporter 4 (MCT4) in NB cells.^22^ The association between metabolism and spontaneous regression of NB is unknown. We wish to know whether metabolism is involved in the process of spontaneous regression.

In this study, we use stage 4s tumours as a surrogate to those NBs underwent spontaneous regression. One RNA‐sequence data sets (n = 153) and two microarray data sets (n = 498 and n = 223) were utilized in this study to perform the analyses. Differentially expressed metabolic genes were identified by comparing those subjects died in stage 4 NB group and those survived in stage 4s NB group. Excluding the dead cases in the stage 4s group would make it better to serve as surrogates to those NBs underwent spontaneous regression. Finally, twelve differentially expressed and survival‐related metabolic genes were incorporated into the metabolic gene signature. Nine metabolism‐related long non‐coding RNAs (lncRNAs) were also identified and incorporated into a metabolism‐related lncRNA signature. The metabolic gene signature and metabolism‐related lncRNA signature performed well in predicting overall survival (OS) of NB patients. Gene set enrichment analysis (GSEA) identifies that multiple metabolic processes are significantly enriched in the high‐risk group, while no metabolic gene set is identified in the low‐risk group. These results suggest that metabolism reprogramming might play important roles in promoting NB progression and thus inhibiting NB regression.

## 2 MATERIALS AND METHODS

### 2.1 Neuroblastoma data set processing

The RNA‐sequence data sets (TARGET NBL, n = 153) were obtained from National Cancer Institute GDC Data Portal. The Agilent microarray data sets GSE49710 (n = 498) were obtained from Gene Expression Omnibus (GEO) database. The Agilent microarray data sets E‐MTAB‐8248 (n = 223) were obtained from ArrayExpress database. The clinical characteristics of the patients in each of the three cohorts are shown in Table S1. The RNA‐sequence data set was used as the discovery cohort and termed as cohort 1. The microarray data sets GSE49710 and E‐MTAB‐8248 were used as the validation cohorts and termed as cohort 2 and cohort 3, respectively. The Agilent microarray probes IDs were firstly annotated using the platform GPL16876 (Agilent‐020382 Human Custom Microarray 44k). Then, to renew the annotation, the probes IDs were re‐annotated according to their corresponding GenBank Accession number. When multiple probes mapped to the same gene, the mean of the signal intensities will be used. The genomic alterations (mutation and copy‐number alteration) of the identified genes were analysed on the open platform of cBio Cancer Genomics Portal (cBioPortal) (http://www.cbioportal.org/).^23^

### 2.2 Extraction of differentially expressed metabolic genes

The KEGG (Kyoto Encyclopedia of Genes and Genomes) gene sets were obtained from Molecular Signatures Database (MSigDB) on gene set enrichment analysis (GSEA) website. All genes included in the KEGG gene sets that associated with metabolism were extracted, a total of 910 genes were identified as involved in metabolism pathways. Differential expression analyses were performed by 'limma' package using the R software. Genes with FDR < 0.05 and |log2FoldChange| > 0.5 were extracted as differentially expressed genes. lncRNAs correlated (Pearson correlation, |r| ≥ 0.3) with metabolic genes were extracted as metabolism‐related lncRNAs. Only those lncRNAs matched to GENCODE annotation of long non‐coding RNA (release 31, GRCh38.p12) were selected.

### 2.3 Construction of the prognostic metabolic gene signatures

Univariate Cox proportional hazards regression analyses were performed to identify those genes associated with overall survival (OS) in the entire cohort 1. A *P‐*value of ≤.05 was considered statistically significant. Those survival‐related genes were put into the least absolute shrinkage and selection operator (LASSO) penalty Cox regression analysis to eliminate false positives because of over‐fitting.^24^ Finally, the prognostic metabolic gene signature was constructed by weighting the Cox regression coefficients to calculate a risk score for each subject. The median value was used as the cut‐off value, and the patients were classified as low‐risk and high‐risk group accordingly. The same formula and the same cut‐off value were applied to the validation cohorts. Metabolism‐related lncRNA signature was constructed by the same method.

### 2.4 Function annotation and gene set enrichment analysis

The bubble plot and circle plot of Gene Ontology (GO) and KEGG pathway annotation were generated by R package 'ggplot2', 'enrichplot' and 'GOplot'. Functional annotation with a *P‐*value < .05 was considered statistically significant. Gene set enrichment analysis (GSEA) comparing low‐risk and high‐risk groups was performed by GSEA software (version 4.0.03).^25^ A nominal *P*‐value < .05 and false discovery rate (FDR) q‐value < .25 were considered statistically significant for GSEAs. Single sample gene set enrichment analysis (ssGSEA) was performed by R package 'GSVA' to assess the immune status of the tumour samples.^26^, ^27^ The immune‐related gene sets used in this study have been reported previously.^28^, ^29^ Estimation of stromal and immune cells infiltrated into the tumour tissues was performed by R package 'ESTIMATE'.^30^

### 2.5 Immunohistochemistry

A human neuroblastoma tissue array (N264001, Bioaitech, China) was utilized for AKR1C1 immunohistochemical (IHC) staining, which was approved by the Ethics Committee of People's Hospital of Xutong County, Henan Province. This study was approved by the Ethics Committee of Tongji Hospital of Huazhong University of Science and Technology and performed in accordance with the ethical standards of the Declaration of Helsinki. The human paraffin‐embedded tissue array contains 22 NB tissue samples (15 cases of stage 1, 2 cases of stage 3 and 5 cases of stage 4). Tissue sections were subjected to IHC analysis using the Avidin‐Biotin Complex (ABC) Vectastain Kit (SP‐9001, ZsgbBio) according to the manufacturer's protocol. The human AKR1C1 antibody (A13004, ABclonal) was used as the primary antibody. The immunostaining of AKR1C1 was evaluated independently by two pathologists who were blinded to all clinical information. The AKR1C1 protein was found to be expressed primarily in the cytoplasm of tumour cells. The evaluation method that has been reported previously is as follows: The cytoplasm staining fraction (CF) was assigned a score of 0 (0%‐5%), 1 (5%‐25%), 2 (26%‐50%), 3 (51%‐75%) or 4 (>75%), and cytoplasm staining intensity (CI) was noted as 0 (negative), 1 (weak), 2 (moderate) and 3 (strong); subsequently, a combined cytoplasm score (CS) was calculated by multiplying CF and CI (range of 0‐12).^31^

### 2.6 Statistical analysis

The univariate and multivariate Cox proportional hazards regression analyses were performed by the R package 'survival'. The LASSO regression analyses were performed by the R package 'glmnet'. The Kaplan‐Meier plots were constructed by R software or GraphPad Prism 5, and the statistical significance was assessed by two‐sided log‐rank test. The time‐dependent receiver operating characteristic (ROC) curves and area under the curve (AUC) analyses were used to evaluate the predictive performance of the prognostic signatures and performed by the R package 'time ROC'. Correlation network was constructed by R package 'corrr' and 'dplyr'. Volcano plot was plotted by the R package 'ggplot2'. Heatmaps and unsupervised clustering were generated either by the R package 'pheatmap' or by TMeV software (Tigr MultiExperiment Viewer, version 4.9.0). The R software version 3.6.2 was utilized in this study for the statistical analyses. All statistical tests were two‐sided, and a *P*‐value < .05 was considered statistically significant.

## 3 RESULTS

### 3.1 Identification of differentially expressed and survival‐related metabolic genes

**Figure 1:**
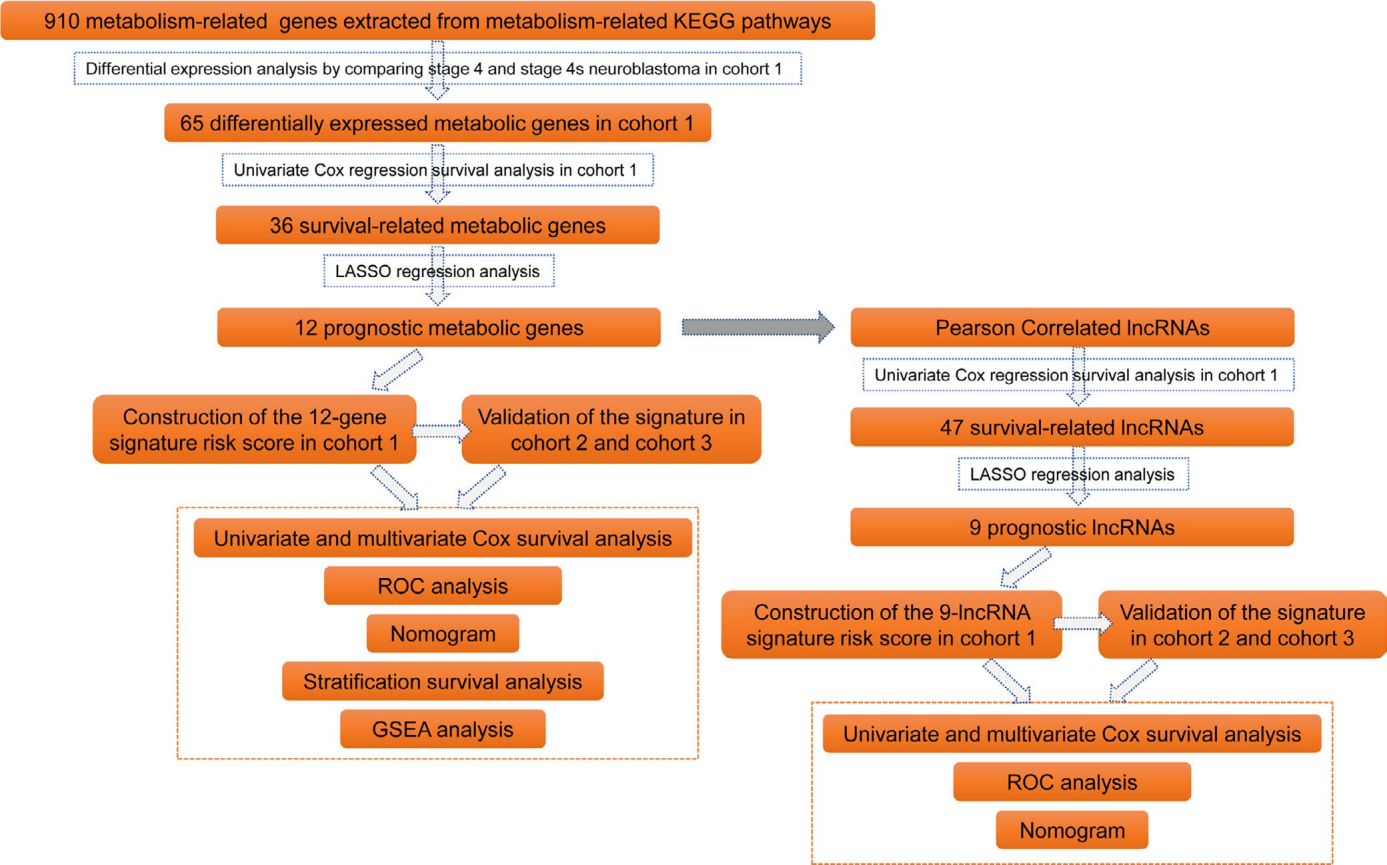
Flow chart of this study

**Figure 2:**
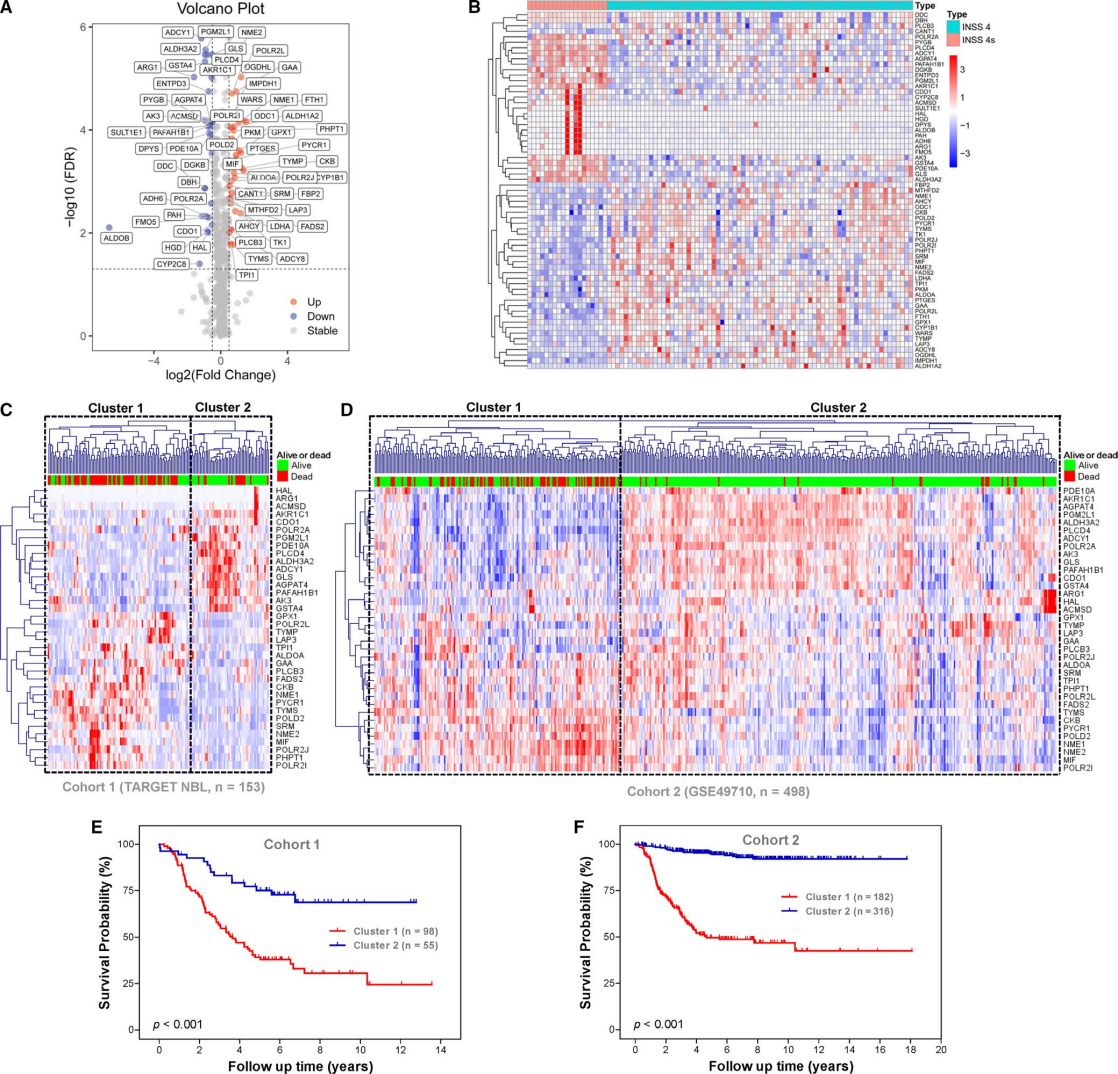
Identification of survival‐related metabolic genes differentially expressed between stage 4s and stage 4 neuroblastomas. A, Volcano plot shows the differentially expressed 65 metabolic genes in cohort 1. B, The heatmap shows the expression pattern of the 65 metabolic genes in cohort 1. C, The 36 survival‐related metabolic genes stratify cohort 1 into two distinct clusters. D, The 36 survival‐related metabolic genes stratify cohort 2 into two distinct clusters. E, Kaplan‐Meier plot for OS of patients in the two clusters of cohort 1. F, Kaplan‐Meier plot for OS of patients in the two clusters of cohort 2

A flow chart of this study is shown in Figure 1. Since most of the subjects in the RNA‐sequence data sets (TARGET NBL) belong to stage 4 or stage 4s disease (Table S1), we decided to first make a comparison between stage 4 and stage 4s NBs in order to find out whether metabolism is associated with spontaneous regression of NB. We excluded those cases who died during follow‐up in stage 4s group to make it better to serve as surrogates to NBs that spontaneously regressed, and also excluded those cases who survived during follow‐up in stage 4 group to make it better to represent those real 'high‐risk' NBs. Finally, 19 cases who survived in stage 4s group and 73 cases who deceased in stage 4 group were used for comparison. The average follow‐up time (OS time) for those survived cases in the stage 4s group (6.04 ± 2.08 years) was relatively longer than that for those deceased cases in the stage 4 group (2.76 ± 1.87 years). A total of 65 metabolic genes were found to be differentially expressed between these two groups in cohort 1. Thirty‐six metabolic genes were up‐regulated in stage 4 NBs, while 29 metabolic genes were up‐regulated in stage 4s NBs (Figure 2A and B).

Univariate survival analyses revealed that 36 metabolic genes were significantly (*P* < .05) associated with OS in entire cohort 1 (Figure S1A). Sixteen of them were up‐regulated in stage 4s NBs and associated with good survival, while twenty were up‐regulated in stage 4 NBs and associated with bad survival.

Unsupervised hierarchical clustering analyses revealed that the 36 survival‐related metabolic genes stratified each of the three cohorts into two distinct clusters (Figures 2C, D and S1B): One cluster has good survival outcome and another cluster has bad survival outcome. In cohort 1, the OS rates at 13 years were 24.40% in cluster 1 compared with 68.78% in cluster 2 (Figure 2E). In cohort 2, the OS rates at 18 years were 42.52% in cluster 1 compared with 92.11% in cluster 2 (Figure 2F). In cohort 3, the OS rates at 19 years were 55.84% in cluster 1 compared with 89.68% in cluster 2 (Figure S1C).

### 3.2 Construction and validation of the prognostic metabolic gene signature

**Figure 3:**
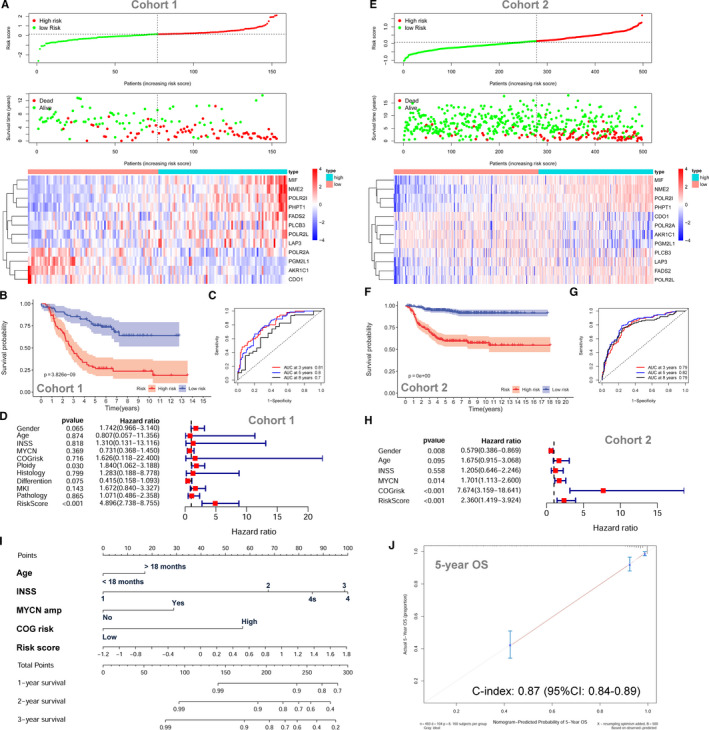
The prognostic role of the metabolic gene signature for neuroblastoma. A, The risk scores distribution, survival status of patients and heatmap of gene expression pattern in cohort 1. B, The Kaplan‐Meier plot for OS of patients in different risk groups of cohort 1. C, Time‐dependent ROC curves for the prognostic value of the metabolic gene signature in cohort 1. D, Multivariate Cox regression survival analysis in cohort 1. E, The risk score distribution, survival status of patients and heatmap of gene expression pattern in cohort 2. F, The Kaplan‐Meier plot for OS of patients in different risk groups of cohort 2. G, Time‐dependent ROC curves for the prognostic value of the metabolic gene signature in cohort 2. H, Multivariate Cox regression survival analysis in cohort 2. I, The nomogram for predicting OS. J, The calibration curve for the nomogram

Twelve metabolic genes were selected by LASSO regression analysis and incorporated into the prognostic gene signature (Figure S1D and E). The characteristics of the 12 metabolism‐related genes are shown in Table S2. The risk scores were calculated for each subject as follows: risk score = 0.0354*FADS2 + 0.0013*POLR2L + 0.0192*MIF + 0.0305*LAP3 + 0.0077*PLCB3 + 0.0029*NME2 + 0.0192*POLR2I + 0.0151*PHPT1 ‐ 0.0227*POLR2A ‐ 0.1016*AKR1C1 ‐ 0.0475*CDO1 ‐ 0.0152*PGM2L1. The entire cohort 1 was classified into two risk groups by the median value of the risk scores. The distribution of risk scores, survival status of each subjects and heatmap of gene expression pattern are shown in Figure 3A. The scatter plot shows that most of the patients in the high‐risk group died and most of the patients in the low‐risk group survived during 13 years of follow‐up (Figure 3A). The heatmap shows that nine metabolic genes are highly expressed in the high‐risk group while only three metabolic genes are highly expressed in the low‐risk group (Figure 3A). The Kaplan‐Meier plot shows that patients in the high‐risk group have a significantly poorer OS than those in the low‐risk group (18.96% vs 63.98%, *P* < .001) (Figure 3B). Time‐dependent ROC curves reveal that the metabolic gene signature has good performance in predicting OS, while the AUC at 3 years, 5 years and 8 years was 0.81, 0.8 and 0.7, respectively (Figure 3C).

The metabolic gene signature is significantly associated with OS in cohort 1 by univariate survival analysis (HR = 4.507; 95% CI: 3.111‐6.529; *P* < .001) (Figure S2A). Multivariate Cox survival analyses including gender (female vs male), age status (<18 months vs ≥18 months), INSS stage (INSS 2/3/4s vs INSS 4), MYCN amplification (non‐amplified vs amplified), COG risk status (low risk vs high risk), ploidy status (hyperploid vs diploid), histology type (favourable vs unfavourable), differentiation (differentiating vs poorly differentiated), MKI (low vs high) and pathology subtype (ganglioneuroblastoma vs neuroblastoma) as covariates were performed. In cohort 1, only the metabolic gene signature (HR = 4.896; 95% CI: 2.738‐8.755, *P* < .001) and ploidy status (HR = 1.840; 95% CI: 1.062‐3.188; *P* = .030) are independently associated with OS by the multivariate model (Figure 3D). The ROC curve analyses reveal that the 5‐year AUC value (0.807) for the metabolic gene signature is higher than all the other risk factors in cohort 1 (Figure S2B).

The metabolic gene signature is tested in cohort 2 and cohort 3 for validation using the same risk score formula and same cut‐off value. The risk distribution, survival status and gene expression pattern of cohort 2 are shown in Figure 3E. The Kaplan‐Meier plot shows that patients in the high‐risk group had a significantly poorer OS than those in the low‐risk group in cohort 2 (54.84% vs 91.52%, *P* < .001) (Figure 3F). The metabolic gene signature has good performance in predicting OS in cohort 2, while the AUC at 3, 5 and 8 years was 0.79, 0.82 and 0.79, respectively (Figure 3G). In cohort 2, the metabolic gene signature risk score is also significantly associated with OS both by univariate model (HR = 7.828; 95% CI: 5.277‐1.611; *P* < .001; Figure S2C) and by multivariate model (HR = 2.360; 95% CI: 1.419‐3.924; *P* < .001; Figure 3H). Consistent with cohort 1 and cohort 2, the validation in cohort 3 shows similar results (Figure S2D‐H). A nomogram including the metabolic gene signature risk score for OS prediction was constructed in cohort 2, which has the largest sample size and consists all different INSS stages (1, 2, 3, 4 and 4s) (Figure 3I). The C‐index for the nomogram is 0.87 (95%: 0.84‐0.89), indicating high prediction accuracy. The calibration curve also revealed that the nomogram‐predicted 5‐year OS was very close to the actual 5‐year OS (Figure 3J).

### 3.3 Prognostic role of the metabolic gene signature within clinical subgroups

**Figure 4:**
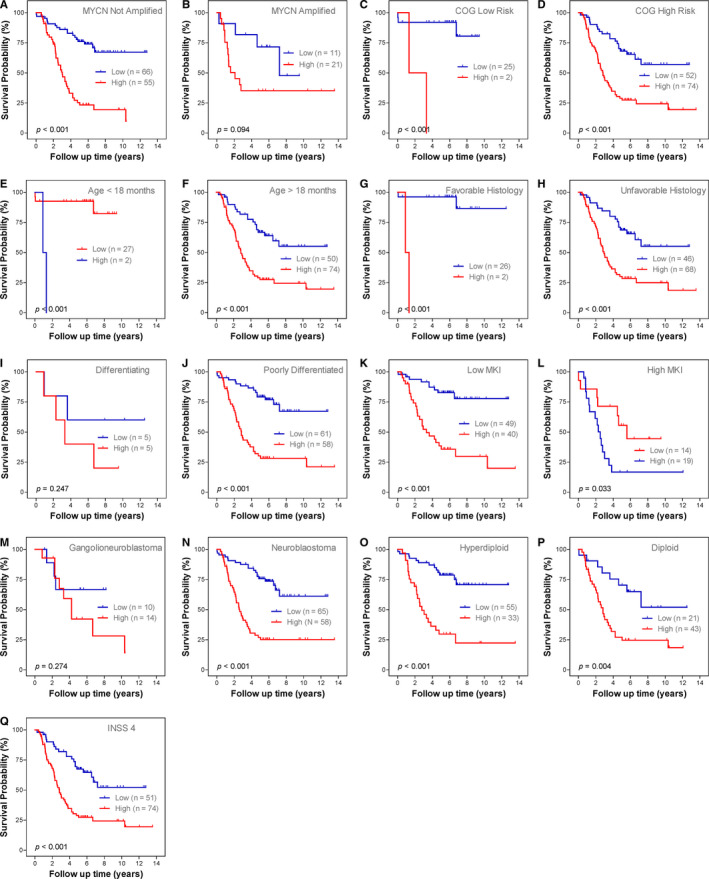
Subgroup Kaplan‐Meier plots for the metabolic gene signature based on different clinical risk factors. A, MYCN not amplified. B, MYCN amplified. C, COG low risk. D, COG high risk. E, Age < 18 mo. F, Age > 18 mo. G, Favourable histology. H, Unfavourable histology. I, Differentiating. J, Poorly differentiated. K, Low MKI. L, High MKI. M, Ganglioneuroblastoma. N, Neuroblastoma. O, Hyperdiploid. P, Diploid. Q, INSS stage 4. The *P*‐values were obtained using a Mantel log‐rank test (two‐sided)

Subgroup Kaplan‐Meier survival analyses were performed based on MYCN amplification status (not amplified and amplified; Figure 4A and B), COG risk status (low and high; Figure 4C and D), age status (age <18 months and age >18 months; Figure 4E and F), histology subtype (favourable and unfavourable; Figure 4G and H), differentiation status (differentiating and poorly differentiated; Figure 4I and J), MKI status (low and high; Figure 4K and L), pathology subtype (ganglioneuroblastoma and neuroblastoma; Figure 4M and N), ploidy status (hyperdiploid and diploid; Figure 4O and P) and INSS stage (stage 4; Figure 4Q). Since only one patient is classified as stage 2, six patients as stage 3 and no patients as stage 1 in cohort 1, the subgroup analysis is not conducted for stage 1, stage 2 and stage 3. All patients in stage 4s were classified as low‐risk group; thus, the Kaplan‐Meier plot is also not constructed.

Within each of these subgroups, patients were grouped into low‐risk and high‐risk groups according to the same cut‐off value as the entire cohort 1. The results show that patients with low‐risk score tend to have a good survival than patients with high‐risk score in each of the subgroup. The survival difference does not reach statistically significant in only three subgroups (MYCN‐amplified subgroup, differentiating subgroup and ganglioneuroblastoma subgroup) with low case number.

### 3.4 Function annotation and GSEA

**Figure 5:**
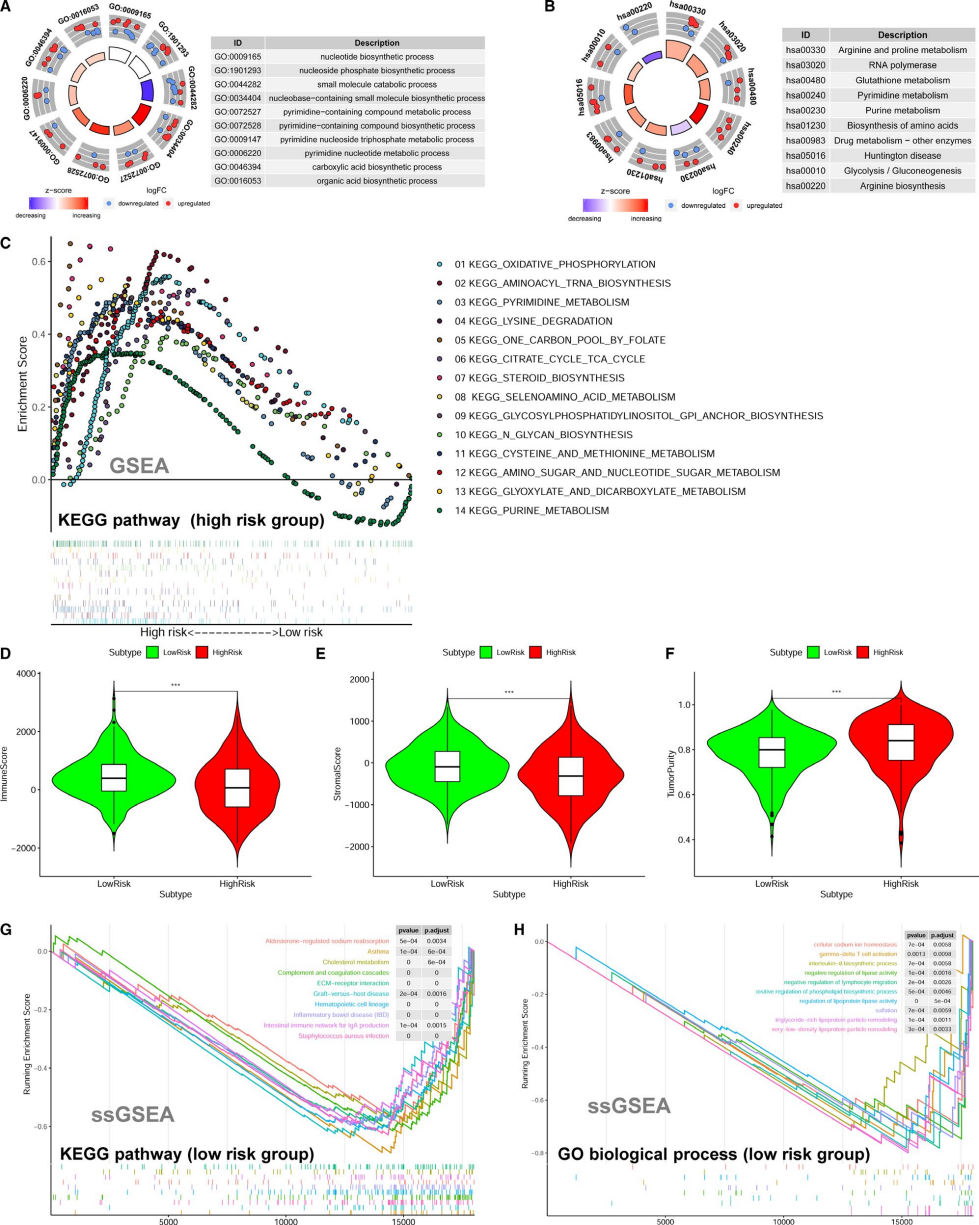
Function annotation and GSEAs for the metabolic gene signature in neuroblastoma. A, The circle plot of GO function annotation for the 36 differentially expressed and survival‐related metabolic genes. B, The circle plot of KEGG pathways for the 36 differentially expressed and survival‐related metabolic genes. C, GSEA shows the KEGG pathways enriched in the high‐risk group of the metabolic gene signature in cohort 2. D, The immune scores of the low‐risk group and high‐risk group. E, The stromal scores of the low‐risk group and high‐risk group. F, The tumour purity of the low‐risk group and high‐risk group. G, The ssGSEA showing the top ranking KEGG pathways enriched in the low‐risk group. H, The ssGSEA showing the top ranking GO biological processes enriched in the low‐risk group. ^***^Indicates *P* < .001

The 36 survival‐related metabolic genes were put into GO and KEGG functional annotation. The bubble plots show the top ranking GO enrichment (Figure S3A) and KEGG pathway enrichment (Figure S3B).The circle plots reveal that most of the metabolic processes of GO (Figure 5A) and metabolic pathways of KEGG (Figure 5B) are up‐regulated in stage 4 NBs.

GSEAs were conducted to compare the low‐risk group with the high‐risk group of the metabolic gene signature in the largest cohort (cohort 2, n = 498). The results show that 14 metabolism‐associated KEGG pathways are significantly enriched in the high‐risk group (Figure 5C). These KEGG pathway gene sets includes oxidative phosphorylation (*P* < .001, FDR = 0.002), aminoacyl tRNA biosynthesis (*P* = .004, FDR = 0.006), pyrimidine metabolism (*P* < .001, FDR = 0.008), lysine degradation (*P* = .009, FDR = 0.012), one‐carbon pool by folate (*P* = .013, FDR = 0.021), citrate cycle TCA cycle (*P* = .014, FDR = 0.020), selenoamino acid metabolism (*P* = .014, FDR = 0.035), glycosylphosphatidylinositol GPI anchor biosynthesis (*P* = .020, FDR = 0.055), N‐glycan biosynthesis (*P* = .038, FDR = 0.054), amino sugar and nucleotide sugar metabolism (*P* = .033, FDR = 0.064), glyoxylate and dicarboxylate metabolism (*P* = .039, FDR = 0.068) and purine metabolism (*P* = .038, FDR = 0.133). No gene set is significantly enriched in the low‐risk group. The top 30 KEGG pathways enriched in the high‐risk and the low‐risk group are shown in Tables S3 and S4, respectively.

Since metabolism is also associated with immune system,^32^, ^33^ we also investigated whether the high‐risk group and the low‐risk group have different status of immune activity. We found that the low‐risk group has a relatively higher level of immune cell infiltrations than the high‐risk group (Figure S3C). The immune score (Figure 5D) and stromal score (Figure 5E) were significantly higher in the low‐risk group than those in the high‐risk group, which indicates a relatively high level of immune activity in the low‐risk group. On the contrary, the level of tumour purity was significantly higher in the high‐risk group than that in the low‐risk group (Figure 5F), indicating a relatively low level of immune cell infiltration in the high‐risk group. The ssGSEAs also revealed that multiple immune‐related KEGG pathways (eg graft‐vs‐host disease and intestinal immune network for IgA production) or GO biological processes (eg gamma‐delta T‐cell activation and interleukin‐8 biosynthetic process) were significantly enriched in the low‐risk group (Figure 5G and H; Tables S6 and S8). Thus, the results of our study also support the link between metabolism and immune activity.

The top 30 KEGG pathways and top 30 GO biological processes enriched in the high‐risk group by the ssGSEAs are shown in Tables S5 and S7, respectively. The KEGG pathway of one‐carbon pool by folate (hsa00670) and oxidative phosphorylation (hsa00190) was also found to be significantly enriched in the high‐risk group by the ssGSEAs (Table S5), indicating that this two metabolism pathways may play important roles in the progression of NB.

### 3.5 Identification of metabolism‐related lncRNAs

Those lncRNAs whose expressions were correlated with the expression of the 12 metabolic genes in the metabolic gene signature were extracted as metabolism‐related lncRNAs. A total of 47 metabolism‐related lncRNAs were shown to be significantly associated with OS by the univariate Cox survival analysis (Figure S4A). Finally, nine survival‐related lncRNAs were selected by LASSO regression analysis and incorporated into the metabolism‐related lncRNA signature (Figure S4B and C). The characteristics of the 9 metabolism‐related lncRNAs are shown in Table S9.

**Figure 6:**
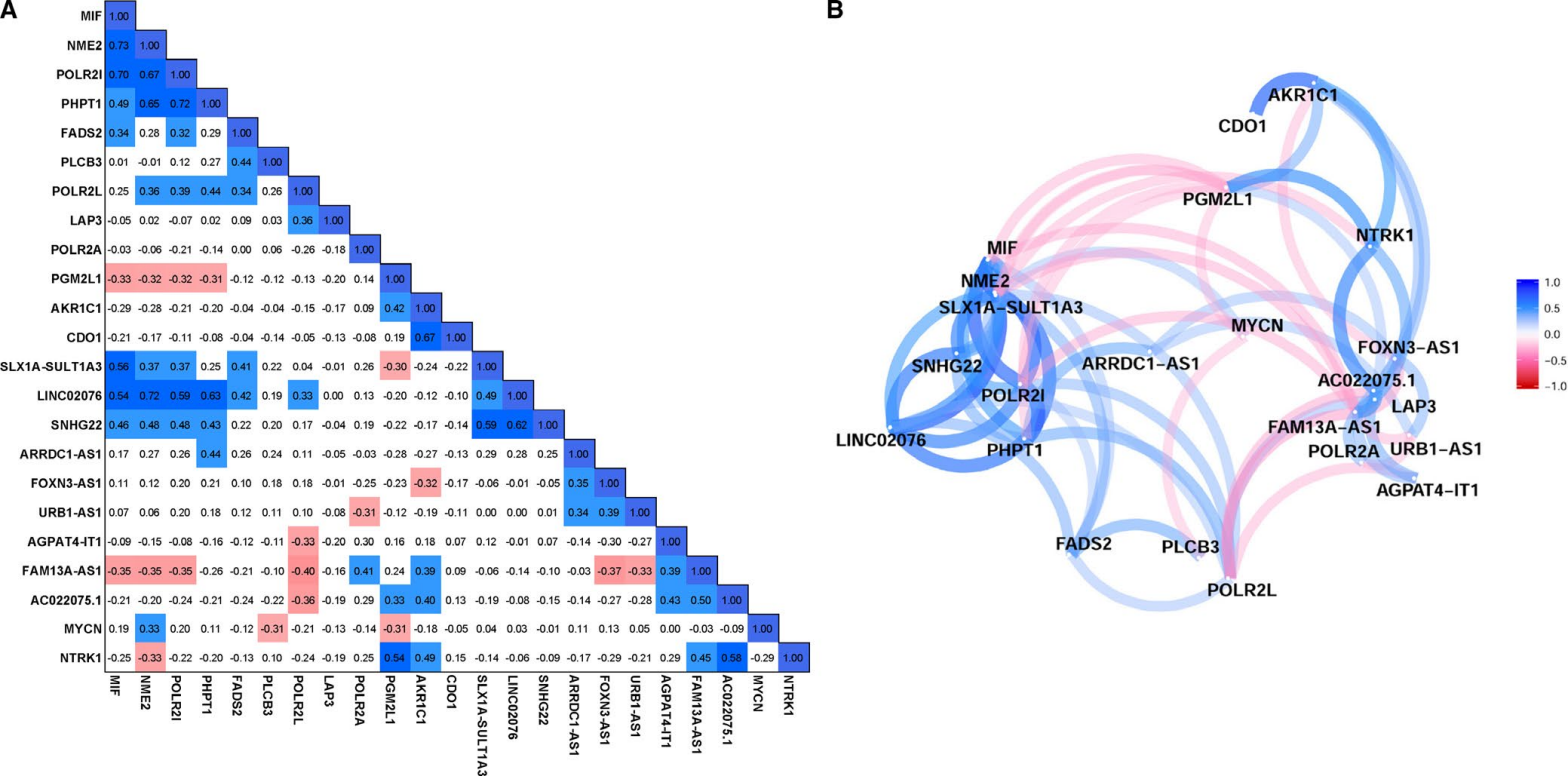
The correlation matrix and network for the metabolic genes and the metabolism‐related lncRNAs. A, Correlation matrix. B, Correlation network. The positive correlation is coloured in blue, and negative correlation is coloured in red

The relationship (Pearson correlation) between these lncRNAs and metabolic genes is shown in Figure 6A and B. Since LASSO regression eliminated most of the co‐linearity issues, only a small of number of these genes were correlated with each other. As MYCN amplification is the most important risk factor for NB and NTRK1 involved in spontaneous regression of NB,^12^, ^34^ we also include MYCN and NTRK1 into the correlation analyses. Only one gene (NME2) is positively correlated with MYCN, and two genes (PLCB3 and PGM2L1) are negatively correlated with MYCN. NME2 is negatively correlated with NTRK1. Two metabolic genes (PGM2L1 and AKR1C1) and two lncRNAs (FAM13A‐AS1 and AC022075.1) are positively correlated with NTRK1.

### 3.6 Construction and validation of metabolism‐related lncRNA signature

**Figure 7:**
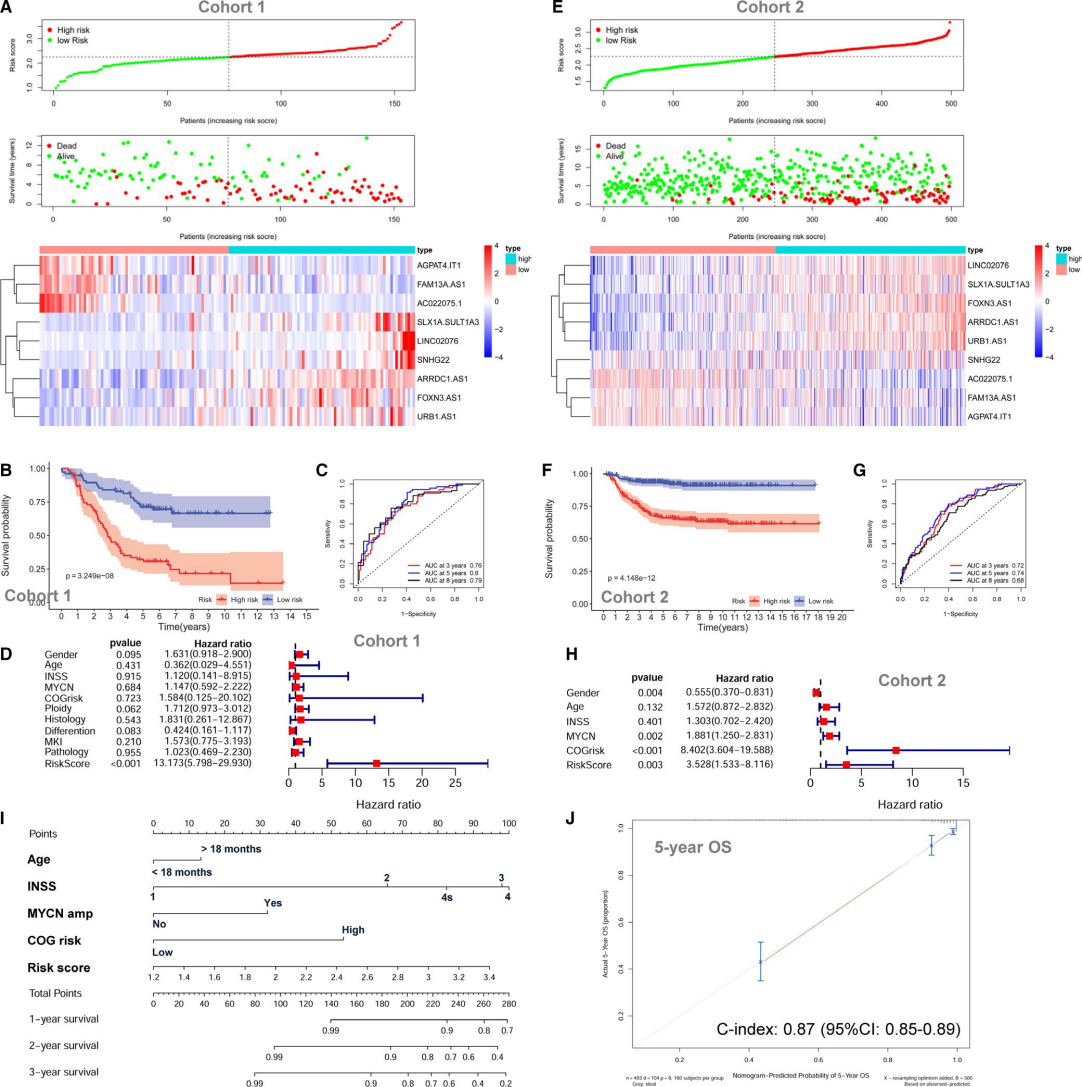
The metabolism‐related lncRNA signature for neuroblastoma. A, The risk score distribution, survival status of patients and heatmap of gene expression pattern in cohort 1. B, The Kaplan‐Meier plot for OS of patients in the different risk groups of cohort 1. C, Time‐dependent ROC curves for the prognostic value of the lncRNA signature in cohort 1. D, Multivariate Cox regression survival analysis in cohort 1. E, The risk score distribution, survival status of patients and heatmap of gene expression pattern in cohort 2. F, The Kaplan‐Meier plot for OS of patients in different risk groups of cohort 2. G, Time‐dependent ROC curves for the prognostic value of the lncRNA signature in cohort 2. H, Multivariate Cox regression survival analysis in cohort 2. I, The nomogram for predicting OS. J, The calibration curve for the nomogram

The lncRNA signature risk scores were calculated for each subject as follows: risk score = 0.03856*FOXN3‐AS1 + 0.1056*LINC02076 + 0.1167*SLX1A‐SULT1A3 + 0.1185*ARRDC1‐AS1 + 0.0195*URB1‐AS1 + 0.0577*SNHG22 ‐ 0.0932*FAM13A‐AS1 ‐ 0.0650*AGPAT4‐IT1 ‐ 0.0600*AC022075.1. The entire cohort 1 was classified into two risk groups by the median value of the risk scores. The risk distribution, survival status and gene expression pattern are shown in Figure 7A. The scatter plot shows that most of the patients in the high‐risk group died and most of the patients in the low‐risk group survived during 13 years of follow‐up (Figure 7A). The heatmap shows that six metabolic lncRNAs were highly expressed in the high‐risk group, while only three metabolic lncRNAs were highly expressed in the low‐risk group (Figure 7A). The Kaplan‐Meier plot shows that patients in the high‐risk group had a significantly poorer OS than patients in the low‐risk group (14.41% vs 66.52%, *P* < .001) (Figure 7B). Time‐dependent ROC curves reveal that the lncRNA signature has good performance in predicting OS in cohort 1, while the AUC at 3, 5 and 8 years was 0.76, 0.8 and 0.79, respectively (Figure 7C). The lncRNA signature risk score is significantly associated with OS both by univariate survival analysis (HR = 6.950; 95% CI: 4.247‐11.407; *P* < .001; Figure S5A) and by multivariate survival analyses adjusted by aforementioned clinical risk factors (HR = 13.173; 95% CI: 5.798‐29.930, *P* < .001; Figure 7D). The ROC curve analyses reveal that the 5‐year AUC value (0.807) of the lncRNA signature is higher than all the other risk factors (Figure S5B).

The lncRNA signature was tested in cohort 2 and cohort 3 for validation. The risk distribution, survival status and gene expression pattern of cohort 2 are shown in Figure 7E. The Kaplan‐Meier plot shows that patients in the high‐risk group have a significantly poorer OS than those in the low‐risk group (61.64% vs 91.03%, *P* < .001) (Figure 7F). The AUC at 3, 5 and 8 years was 0.72, 0.74 and 0.68, respectively (Figure 7G). In cohort 2, the lncRNA signature risk score is also significantly associated with OS both by univariate model (HR = 15.494; 95% CI: 7.793‐30.805; *P* < .001; Figure S5C) and by multivariate model (HR = 3.528; 95% CI: 1.533‐8.116; *P* = .003; Figure 7H). Consistent with cohort 1 and cohort 2, the validation in cohort 3 shows similar results (Figure S5D‐H). A nomogram including the lncRNA signature risk score for OS prediction was constructed in cohort 2, which has the largest sample size and consists all different INSS stages (1, 2, 3, 4 and 4s) (Figure 7I). The C‐index for this nomogram is 0.87 (95%: 0.85‐0.89), which indicates high prediction accuracy. The calibration curve also revealed that the nomogram‐predicted 5‐year OS was very close to the actual 5‐year OS (Figure 7J).

### 3.7 Genetic alterations of the genes in the prognostic signatures

**Figure 8:**
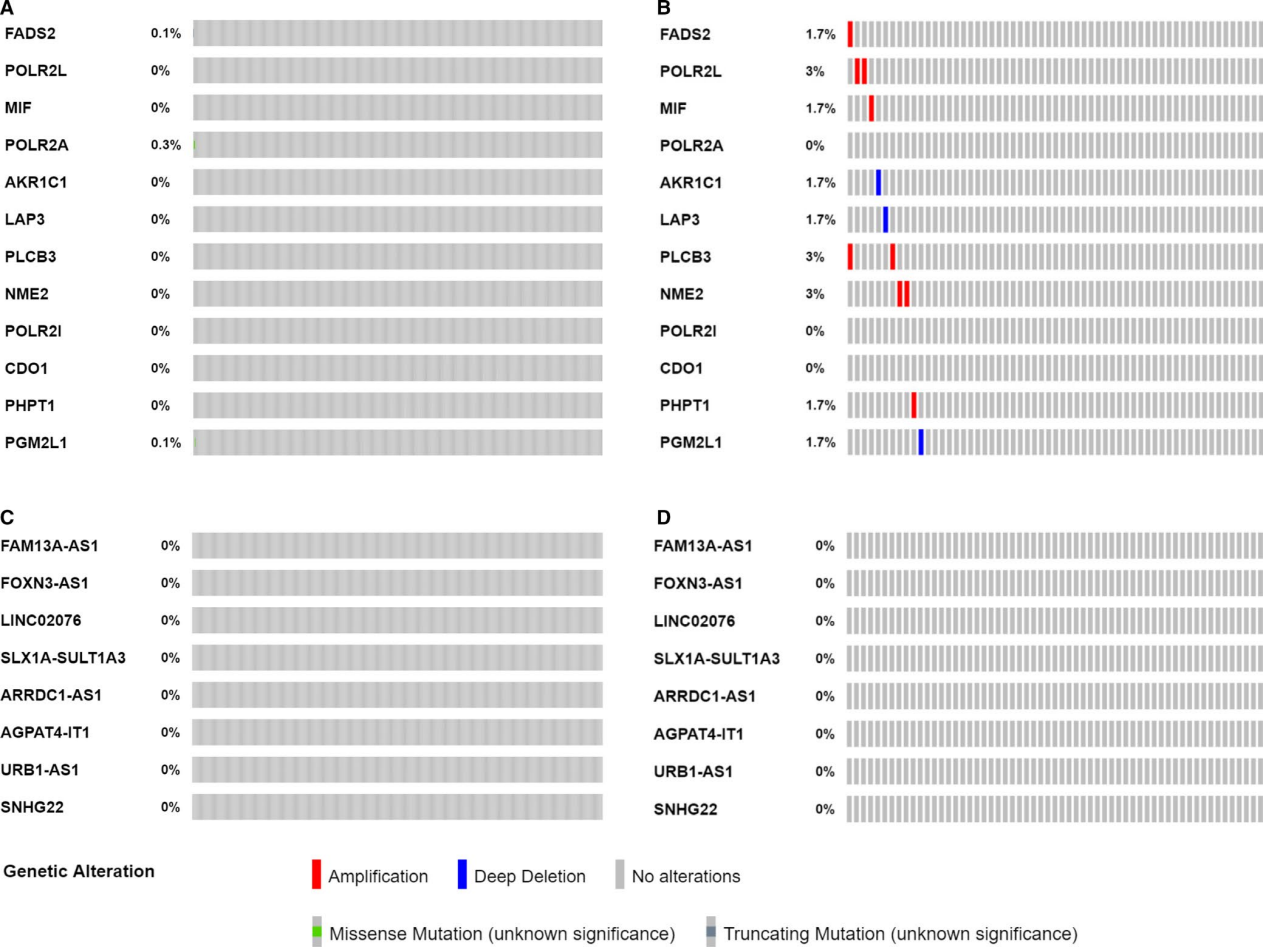
Genetic alterations of the metabolic genes and lncRNAs in neuroblastoma. A, Gene mutation data of the metabolic genes in 755 NB cases. B, Gene copy‐number alteration data of the metabolic genes in 59 NB cases. C, Gene mutation data of the lncRNAs in 755 NB cases. D, Gene copy‐number alteration data of the lncRNAs in 59 NB cases

A total of 755 NB cases with mutation data and 59 NB cases with gene copy‐number data are available in cBioPortal platform. POLR2A is found to have missense mutation in 0.1% of NB cases. FADS2 had missense mutation in 0.1% of NB cases and amplification in 1.7% of NB cases (Figure 8A and B). PGM2L1 had missense mutation in 0.1% of NB cases and deep deletion in 1.7% of NB cases (Figure 8A and B). POLR2L, PLCB3 and NME2 had amplification in 3% of NB cases, respectively (Figure 8B). MIF and PHPT1 had amplification in 1.7% of NB cases, respectively (Figure 8B). AKR1C1 and LAP3 had deep deletion in 1.7% of NB cases, respectively (Figure 8B). No gene alteration data were available for the lncRNA AC0022075.1. No mutation or somatic gene copy‐number alteration was discovered for each of the other lncRNAs (Figure 8C and D).

### 3.8 Immunohistochemistry evaluation of AKR1C1 expression in NB tissues

Since AKR1C1 has the highest weighting coefficient (0.1016) in the metabolic gene prognostic signature, we then decided to perform immunohistochemical (IHC) staining to evaluate its expression level in NB tissue samples. The survival outcome of the subjects of the human NB tissue array (N264001, Bioaitech, China) is not available; thus, we could only make comparisons between different INSS stages. We first evaluated the expression levels of AKR1C1 in the largest microarray data set (cohort 2), and the results showed that higher INSS stages tend to have a relatively lower expression level of AKR1C1 (Figure S6A). The relative expression levels of AKR1C1 in INSS stage 4 NBs were significantly lower than that in stage 1, stage 2, stage 3 and stage 4s NBs, respectively (Figure S6A). IHC analysis of the NB tissue array revealed that the expression of the AKR1C1 protein is significantly higher in stage 1 NB tissues than that in stage 4 NB tissues (Figure S6B‐E).

## 4 DISCUSSION

The association between metabolism and NB progression is largely unknown. To our knowledge, our study is the first aimed at screening for metabolic genes that associated with survival of NB patients through mining of both RNA‐sequence and microarray data. Most of the subjects in the RNA‐sequence data sets (TARGET NBL) belong to stage 4 or stage 4s disease (one stage 2, six stage 3, 125 stage 4 and 21 stage 4s). Thus, we decided to make a comparison between stage 4 and stage 4s NBs. Since spontaneous regression of NB is most evident in patients with stage 4s disease, we hope that our research would also have some enlightening significance for the study of spontaneous regression. We excluded the dead cases in stage 4s to make it better to serve as surrogates for NBs underwent spontaneous regression. Actually, only two out of 21 stage 4s patients died in cohort 1, five out 54 stage 4s patients died in cohort 2, and one out 30 stage 4s patients died in cohort 3 during more than 10 years of follow‐up.

In this study, a total of 36 metabolic genes were found to be differentially expressed and significantly associated with OS of NB patients in cohort 1. Twelve metabolic genes were incorporated into the prognostic signature. The metabolic gene signature successfully stratified each of the cohorts into two different risk groups: The low‐risk group has good survival outcome, and the high‐risk group has bad survival outcome. The metabolic gene signature also performed well in the subgroup survival analyses based on different clinical risk factor stratification. Multivariate survival analyses revealed that the prognostic role of this metabolic gene signature is independent with other clinical risk factors. These results suggest metabolic reprogramming is associated with the progression of NB. This metabolic signature has the potential to be used as a risk factor for risk stratification of NB.

Nine (MIF, NME2, POLR2I, PHPT1, FADS2, PLCB3, POLR2L, LAP3 and POLR2A) of the 12 metabolic genes are highly expressed in the high‐risk group, while only three (PGM2L1, AKR1C1 and CDO1) of them are highly expressed in the low‐risk group. Among these metabolic genes, only PLCB3 has been found to play roles in suppressing neuronal differentiation.^35^ None of the other genes have been reported to be associated with NB. Since AKR1C1 (aldo‐keto reductase family 1 member C1) has the largest weighting coefficient in the prognostic signature, we performed a simple experimental validation for AKR1C1. IHC staining of AKR1C1 protein in NB tissue specimens revealed that higher stage NBs tend to have lower AKR1C1 protein expression levels. This result is consistent with our bioinformatic analysis. AKR1C1 is a member of the aldo/keto reductase (AKR) superfamily, which has also been reported to be involved with multiple malignancies.^36^, ^37^, ^38^ Here, we for the first time identified AKR1C1 also associated with the development of NB, though its exact function in NB is unknown. Another drawback is that the survival outcomes of the patients providing these tumour specimens are unavailable, which hinder further evaluation of the prognostic role of AKR1C1 for NB patients. All in all, the exact roles of these metabolic genes in NB and their underlying mechanisms need to be investigated by further studies.

In recent years, lncRNAs are found to play important roles in the genesis and progression of multiple cancer types including NB.^39^, ^40^, ^41^, ^42^, ^43^, ^44^ In this study, nine metabolism‐related and survival‐related lncRNAs were selected by the LASSO regression analyses and incorporated into the metabolism‐related lncRNA signature risk score. The lncRNA signature also successfully stratified each of the cohorts into two risk groups: The low‐risk group has good survival outcome, and the high‐risk group has bad survival outcome. The lncRNA signature performed well in the subgroup survival analyses and was also shown to be independent with other clinical risk factors.

Six (SLX1A‐SULT1A3, LINC02076, SNHG22, ARRDC1‐AS1, FOXN3‐AS1 and URB1‐AS1) of the nine lncRNAs are highly expressed in the high‐risk group, while only three (AGPAT4‐IT1, FAM13A‐AS1 and AC022075.1) of them are highly expressed in the low‐risk group. Overexpression of SNHG22 has been reported to be associated with poor prognosis of patients with epithelial ovarian carcinoma.^45^ ARRDC1‐AS1 was found to be associated with recurrence of breast cancer.^46^ The function of the other lncRNAs and their relation with cancer have not been reported by the literature. The relation of these lncRNAs with metabolism and their role in NB need to be clarified by further researches.

Investigation of 755 NB cases with mutation data and 59 NB cases with somatic gene copy‐number data discovered that most of the metabolic genes had genetic alterations. It seems that genetic alterations somewhat contribute to their altered expression. However, considering the low level of genetic alterations, the altered expression of these metabolic genes seems largely controlled by other mechanisms, which need to be further clarified.

It is very interesting to find that the GO and KEGG function annotation reveal that most of the metabolism biological processes are up‐regulated in stage 4 NB. In consistent with this finding, the GSEAs also reveal that certain metabolic gene sets are significantly enriched in the high‐risk group. No metabolism gene set is enriched in the low‐risk groups by the GSEA. These results suggest that metabolism reprogramming might contribute to the progression of NB and thus inhibit NB to undergo regression. Undoubtedly, the underlying mechanisms need to be clarified by further investigations.

Oxidative phosphorylation (OXPHOX),^47^ pyrimidine metabolism,^48^ one‐carbon pool by folate,^49^ citrate cycle TCA cycle^50^ and purine metabolism^51^ have all been reported to be associated with cancers. Recent studies found that OXPHOX is up‐regulated in certain cancers, promoting OXPHOS inhibitors to be used to target these cancer subtypes.^47^ In this study, we found that OXPHOS is highly enriched in the high‐risk NB group, which highlighting that OXPHOS inhibitor might also be used for this subtype of NB to improve prognosis. We also found that tricarboxylic acid (TCA) cycle is enriched in high‐risk NBs, indicating that high‐risk NBs rely on the TCA cycle for energy production and macromolecule synthesis. Since TCA cycle is also an emerging target for cancer therapy,^50^ targeting TCA cycle might also be a possible therapy for NB. Further studies are needed to clarify how these enriched metabolic processes contribute to NB progression.

There are also some limitations in our study. Firstly, we did not perform enough experimental studies to corroborate our findings. The specific function of these identified metabolic genes and their underlying mechanisms in NB progression or regression need to be investigated by further experimental studies. Secondly, spontaneous regression of NB is not restricted to stage 4s and not all cases in stage 4s underwent spontaneous regression. However, many other investigators have used stage 4s tumours as a surrogate to investigate the mechanisms responsible for spontaneous regression. Despite these drawbacks, the combination of RNA‐sequence data and microarray data, the large sample size of the three cohorts, and the validation of the findings by two independent cohorts all provide a high level of confidence.

It also has to be mentioned that, during the peer‐review process, one reviewer suggested to combine metabolism‐related coding gene and lncRNA into one prediction model, we did not follow this suggestion due to the following three reasons. Firstly, we initially tried to combine coding gene and lncRNA together and selected out eight genes (MIF, LAP3, FADS2, SNHG22, CDO1, POLR2A, ARRDC1‐AS1 and AC022075.1) to construct one prediction model. However, the prediction ability of the new model was not that good. The new prediction model was not independent with other risk factors when it was tested in both of the two validation cohorts. Secondly, in view of convenient clinical use, it would be better to not combine coding and non‐coding genes together in one prediction model. For the coding genes, we can test them in the tumour tissues by immunohistochemistry, which is usually used in clinical practice. However, for non‐coding genes, we have to resort to other tasting methods. Thirdly, as another reviewer pointed out, the selection of the metabolism‐related lncRNAs is solely based on gene expression correlation with the metabolic coding genes. These lncRNAs are poorly characterized so far, and their actual association with metabolism processes is still need to be investigated and validated by future studies. However, we wish these metabolism‐related lncRNA identified in our study would give some clues to future studies to investigate lncRNAs that are associated with metabolism in NB. Indeed, for a better clinical use, these prediction models also need to be validated and improved by further researches.

In conclusion, we find that metabolic genes are differentially expressed between the stage 4 and stage 4s NB samples. The metabolic gene signature has good performance in predicting OS of NB patients. The metabolism‐related lncRNA signature also has good performance in predicting OS of NB patients. The prognostic value of both the metabolic gene signature and metabolism‐related lncRNA signature is independent with other clinical risk factors. Therefore, the metabolic signatures have the potential to be used for risk stratification of NB. Multiple metabolic processes are enriched in high‐risk NB, indicating that metabolic reprogramming is associated with the progression of NB. Targeting certain metabolic pathways might also be a promising therapy for NB.

## CONFLICT OF INTEREST

The authors confirm that there are no conflicts of interest.

## AUTHOR CONTRIBUTION

**Xinyao Meng:** Data curation (equal); Formal analysis (equal); Investigation (equal); Software (equal); Visualization (equal); Writing‐original draft (equal). **Chenzhao Feng:** Data curation (equal); Formal analysis (equal); Investigation (equal); Software (equal); Visualization (equal); Writing‐original draft (equal). **Erhu Fang:** Formal analysis (supporting); Investigation (supporting); Software (supporting). **Jiexiong Feng:** Funding acquisition (supporting); Project administration (supporting); Writing‐review & editing (supporting). **Xiang Zhao:** Conceptualization (lead); Formal analysis (supporting); Funding acquisition (lead); Methodology (lead); Project administration (lead); Resources (lead); Software (supporting); Supervision (lead); Validation (lead); Writing‐review & editing (lead).

## Supporting information

Supplementary Material

## Data Availability

The data set TARGET NBL is public available and can be found in the National Cancer Institute GDC Data Portal [https://portal.gdc.cancer.gov]. The data set GEO49710 is publicly available and can be found in the GEO data sets [https://www.ncbi.nlm.nih.gov/geo/query/acc.cgi?acc=GSE49710]. The data set E‐MTAB‐8248 is publicly available and can be found in ArrayExpress [https://www.ebi.ac.uk/arrayexpress/experiments/E‐MTAB‐8248/]. All other data analysed during this study are included in the manuscript.

## References

[1] PDQ Pediatric Treatment Editorial Board . Neuroblastoma Treatment (PDQ(R)): Health Professional Version In: PDQ Cancer Information Summaries. . Bethesda (MD): National Cancer Institute (US); 2002.

[2] Gurney JG , Ross JA , Wall DA , Bleyer WA , Severson RK , Robison LL . Infant cancer in the U.S.: histology‐specific incidence and trends, 1973 to 1992. J Pediatr Hematol Oncol. 1997;19(5):428‐432. 9329464 10.1097/00043426-199709000-00004

[3] London WB , Castleberry RP , Matthay KK , et al. Evidence for an age cutoff greater than 365 days for neuroblastoma risk group stratification in the Children's Oncology Group. J Clin Oncol. 2005;23(27):6459‐6465. 16116153 10.1200/JCO.2005.05.571

[4] Maris JM . Recent advances in neuroblastoma. N Engl J Med. 2010;362(23):2202‐2211. 20558371 10.1056/NEJMra0804577 PMC3306838

[5] Evans AE , D'Angio GJ , Randolph J . A proposed staging for children with neuroblastoma. Children's cancer study group A. Cancer. 1971;27(2):374‐378. 5100400 10.1002/1097-0142(197102)27:2<374::aid-cncr2820270221>3.0.co;2-g

[6] Brodeur GM , Pritchard J , Berthold F , et al. Revisions of the international criteria for neuroblastoma diagnosis, staging, and response to treatment. J Clin Oncol. 1993;11(8):1466‐1477. 8336186 10.1200/JCO.1993.11.8.1466

[7] Cohn SL , Pearson AD , London WB , et al. The International Neuroblastoma Risk Group (INRG) classification system: an INRG Task Force report. J Clin Oncol. 2009;27(2):289‐297. 19047291 10.1200/JCO.2008.16.6785 PMC2650388

[8] Suita S , Zaizen Y , Sera Y , et al. Mass screening for neuroblastoma: quo vadis? A 9‐year experience from the Pediatric Oncology Study Group of the Kyushu area in Japan. J Pediatr Surg. 1996;31(4):555‐558. 8801312 10.1016/s0022-3468(96)90495-9

[9] Bessho F . Comparison of the incidences of neuroblastoma for screened and unscreened cohorts. Acta Paediatr. 1999;88(4):404‐406. 10342538 10.1080/08035259950169774

[10] Woods WG , Gao RN , Shuster JJ , et al. Screening of infants and mortality due to neuroblastoma. N Engl J Med. 2002;346(14):1041‐1046. 11932470 10.1056/NEJMoa012387

[11] Erttmann R , Tafese T , Berthold F , et al. 10 years' neuroblastoma screening in Europe: preliminary results of a clinical and biological review from the Study Group for Evaluation of Neuroblastoma Screening in Europe (SENSE). Eur J Cancer. 1998;34(9):1391‐1397. 9849422 10.1016/s0959-8049(98)00135-x

[12] Brodeur GM . Spontaneous regression of neuroblastoma. Cell Tissue Res. 2018;372(2):277‐286. 29305654 10.1007/s00441-017-2761-2 PMC5920563

[13] Lavarino C , Cheung NK , Garcia I , et al. Specific gene expression profiles and chromosomal abnormalities are associated with infant disseminated neuroblastoma. BMC Cancer. 2009;9:44. 19192278 10.1186/1471-2407-9-44 PMC2642835

[14] Yu F , Zhu X , Feng C , et al. Proteomics‐based identification of spontaneous regression‐associated proteins in neuroblastoma. J Pediatr Surg. 2011;46(10):1948‐1955. 22008333 10.1016/j.jpedsurg.2011.06.024

[15] Benard J , Raguenez G , Kauffmann A , et al. MYCN‐non‐amplified metastatic neuroblastoma with good prognosis and spontaneous regression: a molecular portrait of stage 4S. Mol Oncol. 2008;2(3):261‐271. 19383347 10.1016/j.molonc.2008.07.002 PMC5527812

[16] Vander Heiden MG , DeBerardinis RJ . Understanding the Intersections between Metabolism and Cancer Biology. Cell. 2017;168(4):657‐669. 28187287 10.1016/j.cell.2016.12.039 PMC5329766

[17] Kreuzaler P , Panina Y , Segal J , Yuneva M . Adapt and conquer: Metabolic flexibility in cancer growth, invasion and evasion. Mol Metab. 2020;33:83‐101. 31668988 10.1016/j.molmet.2019.08.021 PMC7056924

[18] Payen VL , Mina E , Van Hee VF , Porporato PE , Sonveaux P . Monocarboxylate transporters in cancer. Mol Metab. 2020;33:48‐66. 31395464 10.1016/j.molmet.2019.07.006 PMC7056923

[19] Garcia‐Bermudez J , Williams RT , Guarecuco R , Birsoy K . Targeting extracellular nutrient dependencies of cancer cells. Mol Metab. 2020;33:67‐82. 31926876 10.1016/j.molmet.2019.11.011 PMC7056928

[20] Lacroix M , Riscal R , Arena G , Linares LK , Le Cam L . Metabolic functions of the tumor suppressor p53: Implications in normal physiology, metabolic disorders, and cancer. Mol Metab. 2020;33:2‐22. 31685430 10.1016/j.molmet.2019.10.002 PMC7056927

[21] Wang T , Liu L , Chen X , et al. MYCN drives glutaminolysis in neuroblastoma and confers sensitivity to an ROS augmenting agent. Cell Death Dis. 2018;9(2):220. 29445162 10.1038/s41419-018-0295-5 PMC5833827

[22] Khan A , Valli E , Lam H , et al. Targeting metabolic activity in high‐risk neuroblastoma through Monocarboxylate Transporter 1 (MCT1) inhibition. Oncogene. 2020;39(17):3555‐3570. 32123312 10.1038/s41388-020-1235-2 PMC7970707

[23] Cerami E , Gao J , Dogrusoz U , et al. The cBio cancer genomics portal: an open platform for exploring multidimensional cancer genomics data. Cancer Discov. 2012;2(5):401‐404. 22588877 10.1158/2159-8290.CD-12-0095 PMC3956037

[24] Tibshirani R . The lasso method for variable selection in the Cox model. Stat Med. 1997;16(4):385‐395. 9044528 10.1002/(sici)1097-0258(19970228)16:4<385::aid-sim380>3.0.co;2-3

[25] Subramanian A , Tamayo P , Mootha VK , et al. Gene set enrichment analysis: a knowledge‐based approach for interpreting genome‐wide expression profiles. Proc Natl Acad Sci USA. 2005;102(43):15545‐15550. 16199517 10.1073/pnas.0506580102 PMC1239896

[26] Barbie DA , Tamayo P , Boehm JS , et al. Systematic RNA interference reveals that oncogenic KRAS‐driven cancers require TBK1. Nature. 2009;462(7269):108‐112. 19847166 10.1038/nature08460 PMC2783335

[27] Hänzelmann S , Castelo R , Guinney J . GSVA: gene set variation analysis for microarray and RNA‐seq data. BMC Bioinform. 2013;14:7. 10.1186/1471-2105-14-7 PMC3618321 23323831

[28] Charoentong P , Finotello F , Angelova M , et al. Pan‐cancer immunogenomic analyses reveal genotype‐immunophenotype relationships and predictors of response to checkpoint blockade. Cell Rep. 2017;18(1):248‐262. 28052254 10.1016/j.celrep.2016.12.019

[29] He Y , Jiang Z , Chen C , Wang X . Classification of triple‐negative breast cancers based on Immunogenomic profiling. J Exp Clin Cancer Res. 2018;37(1):327. 30594216 10.1186/s13046-018-1002-1 PMC6310928

[30] Yoshihara K , Shahmoradgoli M , Martínez E , et al. Inferring tumour purity and stromal and immune cell admixture from expression data. Nat Commun. 2013;4:2612. 24113773 10.1038/ncomms3612 PMC3826632

[31] Scharl A , Vierbuchen M , Conradt B , Moll W , Würz H , Bolte A . Immunohistochemical detection of progesterone receptor in formalin‐fixed and paraffin‐embedded breast cancer tissue using a monoclonal antibody. Arch Gynecol Obstet. 1990;247(2):63‐71. 2350195 10.1007/BF02390663

[32] Domínguez‐Andrés J , Joosten LA , Netea MG . Induction of innate immune memory: the role of cellular metabolism. Curr Opin Immunol. 2019;56:10‐16. 30240964 10.1016/j.coi.2018.09.001

[33] Andrejeva G , Rathmell JC . Similarities and distinctions of cancer and immune metabolism in inflammation and tumors. Cell Metab. 2017;26(1):49‐70. 28683294 10.1016/j.cmet.2017.06.004 PMC5555084

[34] Brodeur GM , Minturn JE , Ho R , et al. Trk receptor expression and inhibition in neuroblastomas. Clin Cancer Res. 2009;15(10):3244‐3250. 19417027 10.1158/1078-0432.CCR-08-1815 PMC4238907

[35] Novak JE , Agranoff BW , Fisher SK . Increased expression of Galpha(q/11) and of phospholipase‐Cbeta1/4 in differentiated human NT2‐N neurons: enhancement of phosphoinositide hydrolysis. J Neurochem. 2000;74(6):2322‐2330. 10820192 10.1046/j.1471-4159.2000.0742322.x

[36] Zhu H , Chang L‐L , Yan F‐J , et al. AKR1C1 activates STAT3 to promote the metastasis of non‐small cell lung cancer. Theranostics. 2018;8(3):676‐692. 29344298 10.7150/thno.21463 PMC5771085

[37] Huebbers CU , Verhees F , Poluschkin L , et al. Upregulation of AKR1C1 and AKR1C3 expression in OPSCC with integrated HPV16 and HPV‐negative tumors is an indicator of poor prognosis. Int J Cancer. 2019;144(10):2465‐2477. 30367463 10.1002/ijc.31954

[38] Yun H , Xie J , Olumi AF , Ghosh R , Kumar AP . Activation of AKR1C1/ERβ induces apoptosis by downregulation of c‐FLIP in prostate cancer cells: A prospective therapeutic opportunity. Oncotarget. 2015;6(13):11600‐11613. 25816367 10.18632/oncotarget.3417 PMC4484479

[39] Ma L , Bajic VB , Zhang Z . On the classification of long non‐coding RNAs. RNA Biol. 2013;10(6):925‐933. 23696037 10.4161/rna.24604 PMC4111732

[40] Bhan A , Soleimani M , Mandal SS . Long noncoding RNA and cancer: a new paradigm. Cancer Res. 2017;77(15):3965‐3981. 28701486 10.1158/0008-5472.CAN-16-2634 PMC8330958

[41] Tang Y , Cheung BB , Atmadibrata B , et al. The regulatory role of long noncoding RNAs in cancer. Cancer Lett. 2017;391:12‐19. 28111137 10.1016/j.canlet.2017.01.010

[42] Russell MR , Penikis A , Oldridge DA , et al. CASC15‐S is a tumor suppressor lncRNA at the 6p22 neuroblastoma susceptibility locus. Cancer Res. 2015;75(15):3155‐3166. 26100672 10.1158/0008-5472.CAN-14-3613 PMC4526355

[43] Li D , Wang X , Mei H , et al. Long noncoding RNA pancEts‐1 promotes neuroblastoma progression through hnRNPK‐mediated beta‐catenin stabilization. Cancer Res. 2018;78(5):1169‐1183. 29311158 10.1158/0008-5472.CAN-17-2295

[44] Liu PY , Tee AE , Milazzo G , et al. The long noncoding RNA lncNB1 promotes tumorigenesis by interacting with ribosomal protein RPL35. Nat Commun. 2019;10(1):5026. 31690716 10.1038/s41467-019-12971-3 PMC6831662

[45] Zhang PF , Wu J , Luo JH , et al. SNHG22 overexpression indicates poor prognosis and induces chemotherapy resistance via the miR‐2467/Gal‐1 signaling pathway in epithelial ovarian carcinoma. Aging (Albany NY). 2019;11(19):8204‐8216. 31581131 10.18632/aging.102313 PMC6814594

[46] Liu H , Li J , Koirala P , et al. Long non‐coding RNAs as prognostic markers in human breast cancer. Oncotarget. 2016;7(15):20584‐20596. 26942882 10.18632/oncotarget.7828 PMC4991477

[47] Ashton TM , McKenna WG , Kunz‐Schughart LA , Higgins GS . Oxidative phosphorylation as an emerging target in cancer therapy. Clin Cancer Res. 2018;24(11):2482‐2490. 29420223 10.1158/1078-0432.CCR-17-3070

[48] Parker WB . Enzymology of purine and pyrimidine antimetabolites used in the treatment of cancer. Chem Rev. 2009;109(7):2880‐2893. 19476376 10.1021/cr900028p PMC2827868

[49] Newman AC , Maddocks ODK . One‐carbon metabolism in cancer. Br J Cancer. 2017;116(12):1499‐1504. 28472819 10.1038/bjc.2017.118 PMC5518849

[50] Anderson NM , Mucka P , Kern JG , Feng H . The emerging role and targetability of the TCA cycle in cancer metabolism. Protein Cell. 2018;9(2):216‐237. 28748451 10.1007/s13238-017-0451-1 PMC5818369

[51] Yin J , Ren W , Huang X , Deng J , Li T , Yin Y . Potential mechanisms connecting purine metabolism and cancer therapy. Front Immunol. 2018;9:1697. 30105018 10.3389/fimmu.2018.01697 PMC6077182

