# Supplementary material for: Combined analysis of RNA‐sequence and microarray data reveals effective metabolism‐based prognostic signature for neuroblastoma
Source: J Cell Mol Med. 2020 Jul 19;24(18):10367–81. doi: 10.1111/jcmm.15650 (PMC7521294; doi:10.1111/jcmm.15650)
Supplement: Supplementary file 1 — Supplementary Material [file JCMM-24-10367-s001.docx]

Supporting Information

# Supplementary Data

## Supplementary Figures


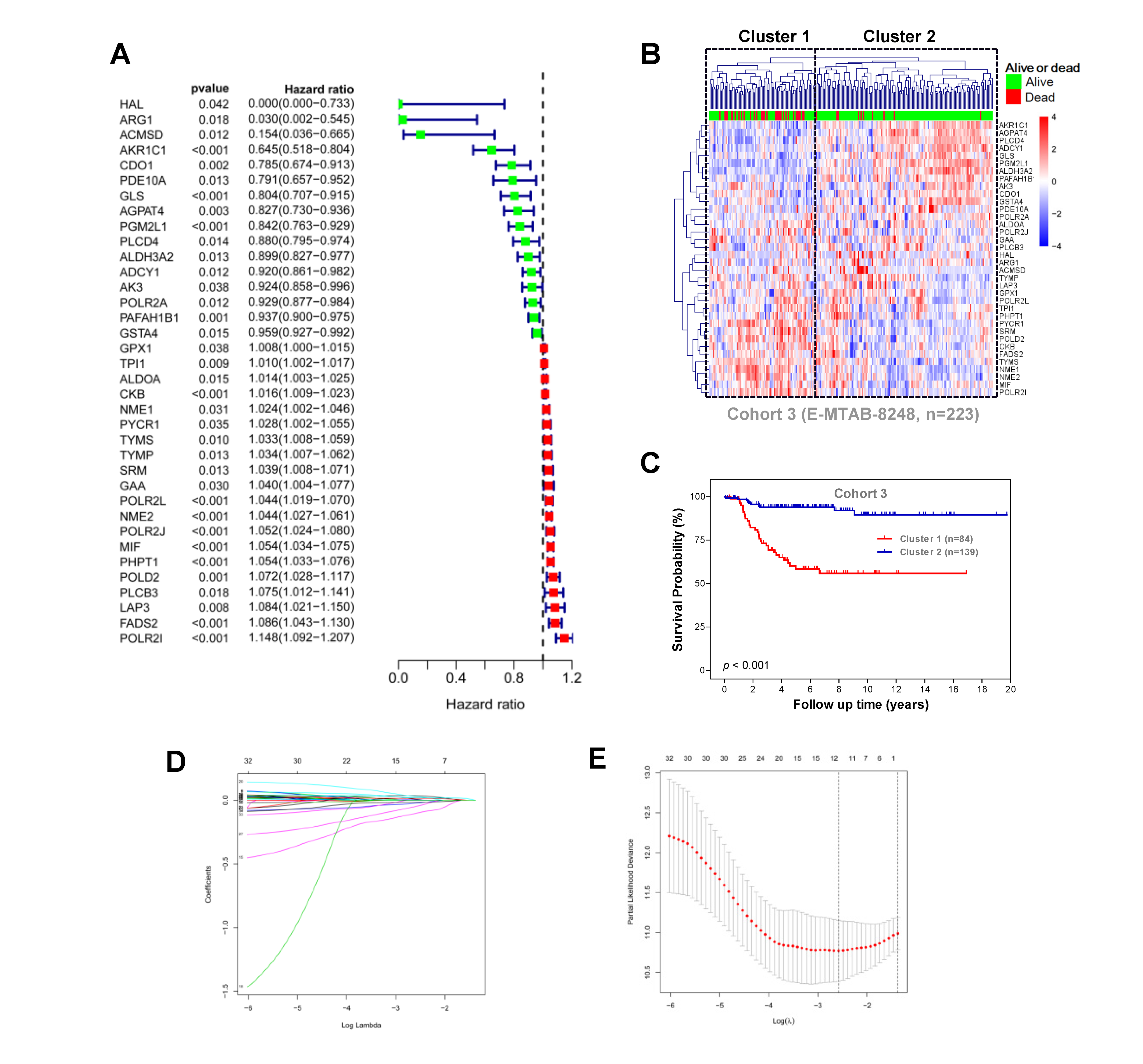


**Figure S1.**The survival-related metabolic genes and LASSO regression analysis. **(A)** The univariate Cox survival analyses for the 36 differentially expressed and survival-related metabolic genes. **(B)** The 36 survival-related metabolic genes stratify cohort 3 into two distinct clusters. **(C)** The Kaplan-Meier plot for OS of patients in the two clusters of cohort 3. **(D, E)** The LASSO regression analysis identified 12 metabolic genes with the best prognostic value to incorporate into the prognostic signature.

**
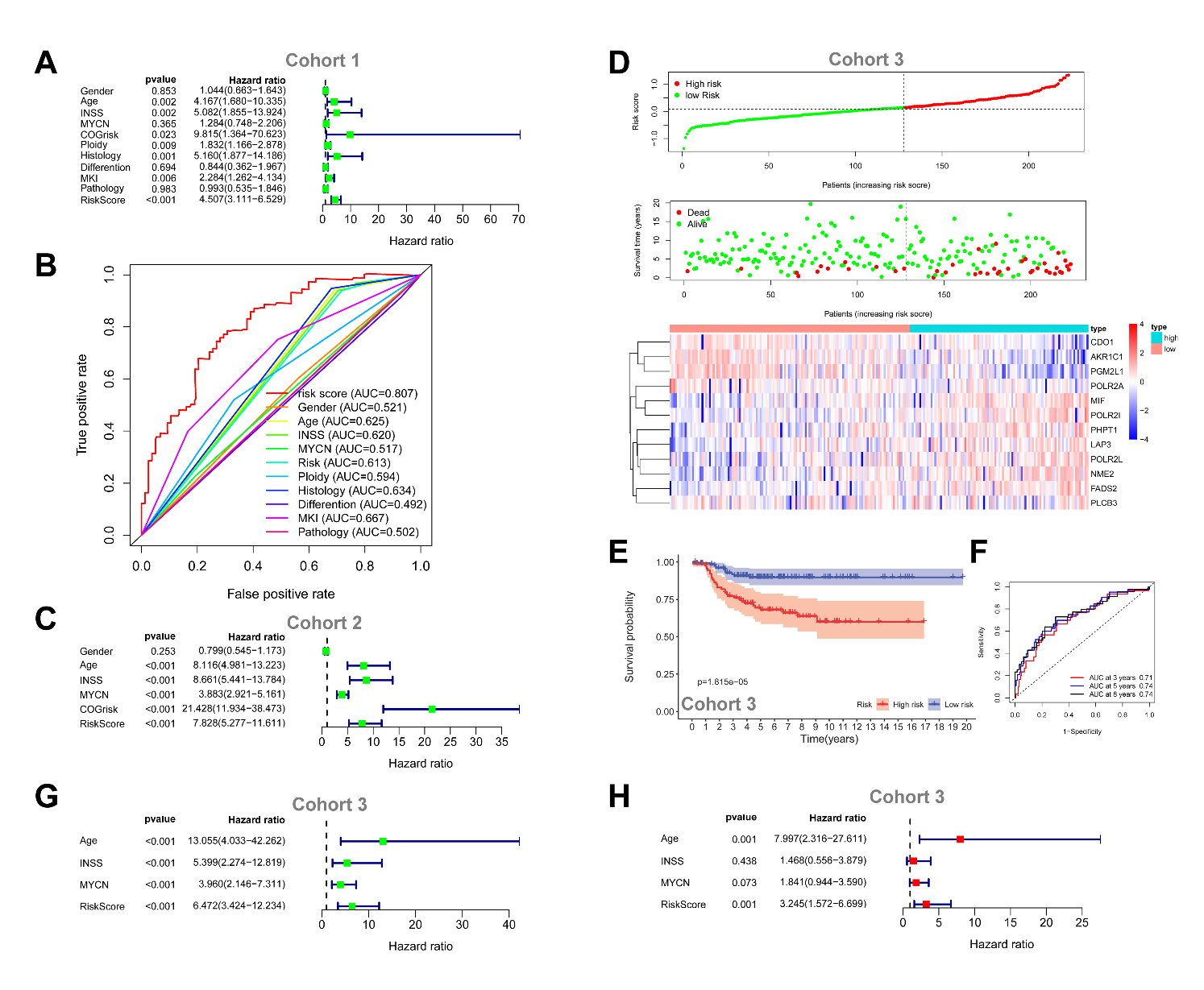
**

**Figure S2.** The prognostic value of the metabolic gene signature. **(A)** The univariate Cox regression survival analysis in cohort 1. **(B)** The ROC curves for the metabolic gene signature and other clinical risk factors in cohort 1. **(C)** The univariate Cox regression survival analysis in cohort 2. **(D)** The risk scores distribution, survival status of patients, and heatmap of genes expression pattern in cohort 3. **(E)** Kaplan-Meier plot for OS of patients in different risk groups of cohort 3. **(F)** Time-dependent ROC curves for the prognostic value of the metabolic gene signature in cohort 3. **(G)** The univariate Cox regression survival analysis in cohort 3. **(H)** The multivariate Cox regression survival analysis in cohort 3.

**
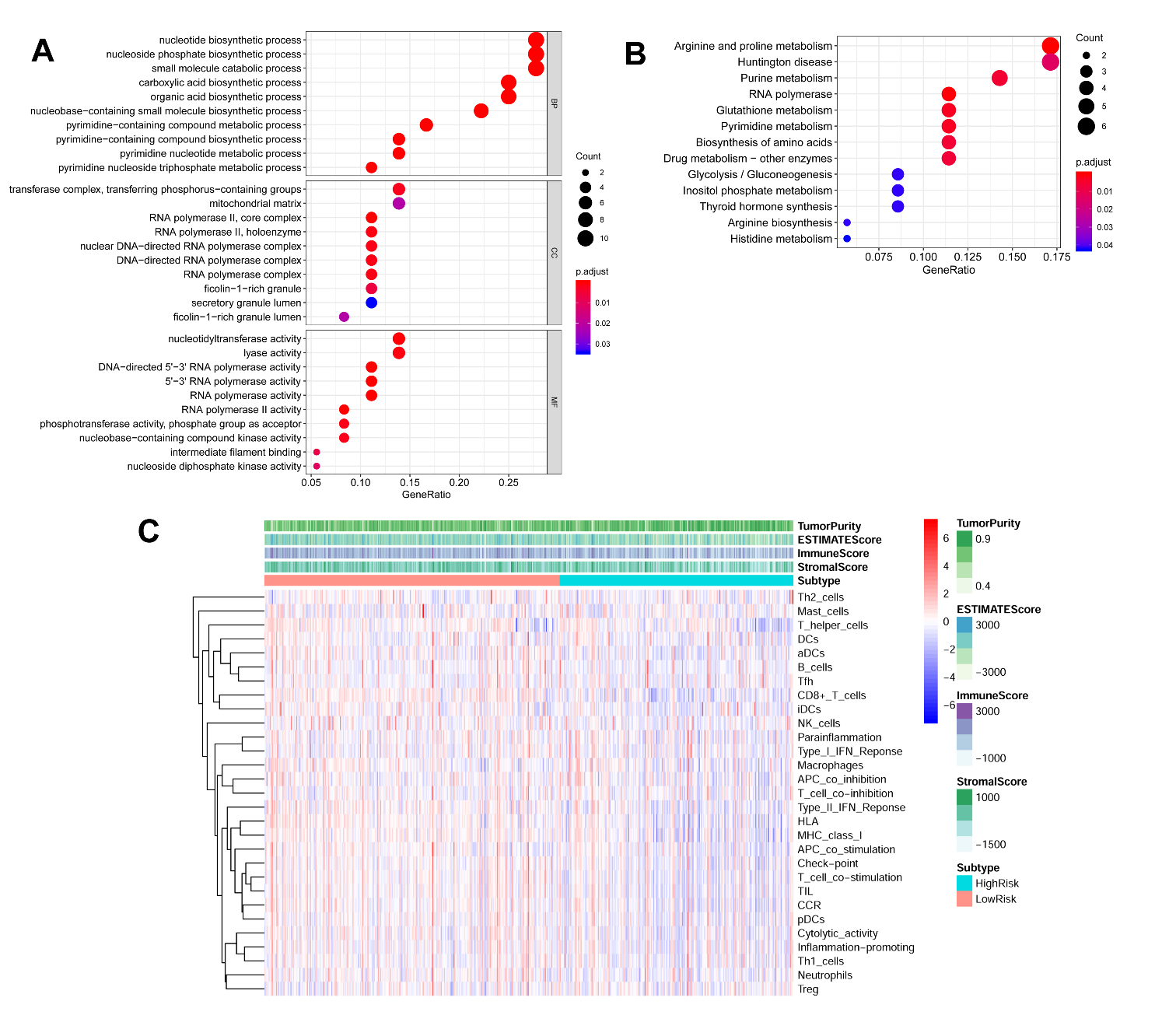
**

**Figure S3. (A)** The bubble plot of GO function annotation for the 36 differentially expressed and survival-related metabolic genes. **(B)** The bubble plot of KEGG pathways for the 36 differentially expressed and survival-related metabolic genes. **(C)** The heatmap showing the immune cells infiltrations in the high risk group and low risk group.


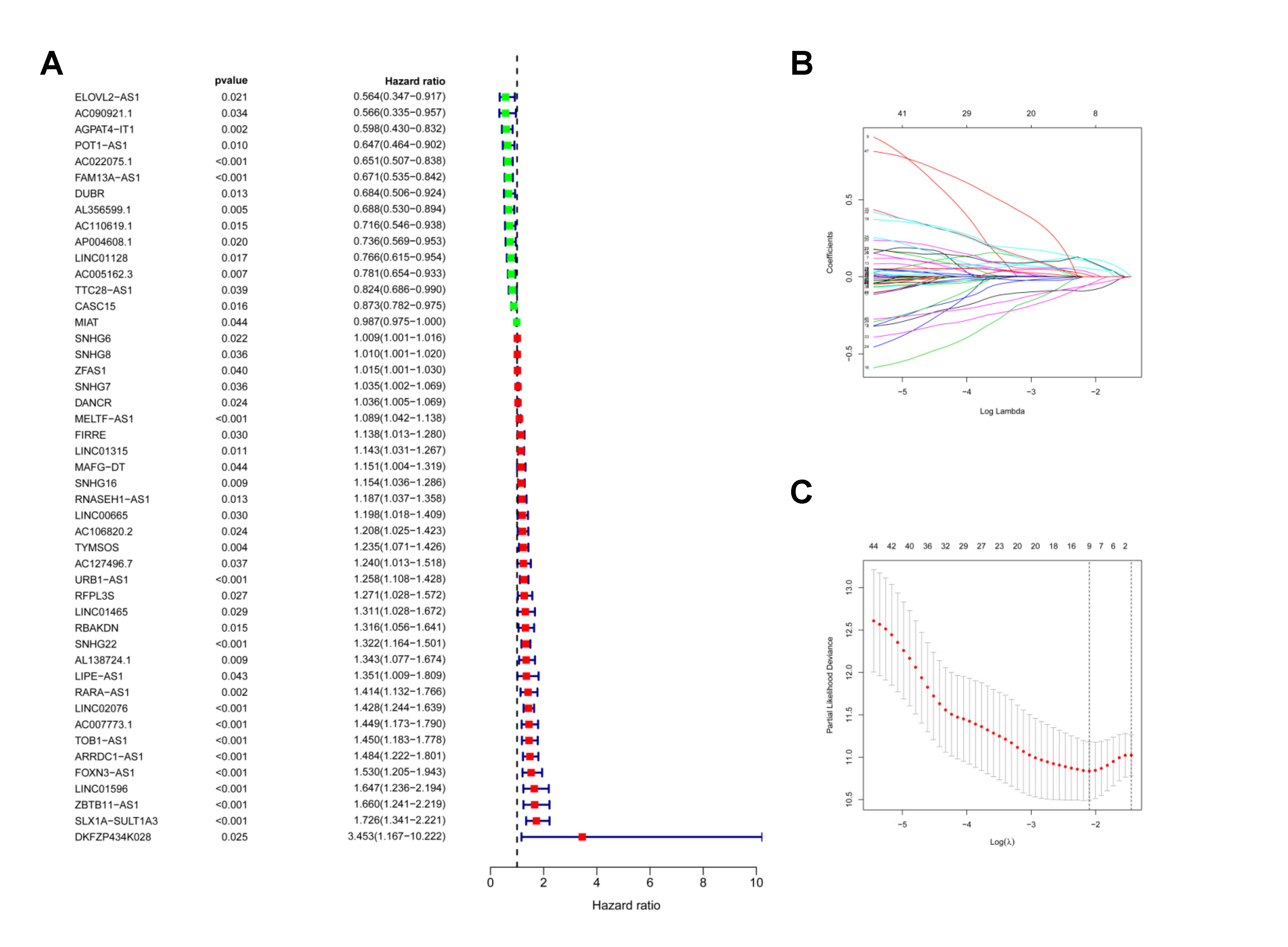


**Figure S4.** The univariate and LASSO regression survival analyses for the metabolism-related lncRNAs in cohort 1. **(A)** The univariate Cox survival analyses for the 47 survival-related lncRNAs. **(B, C)** The LASSO regression analysis identified nine lncRNAs with the best prognostic value to incorporate into the prognostic signature.

**
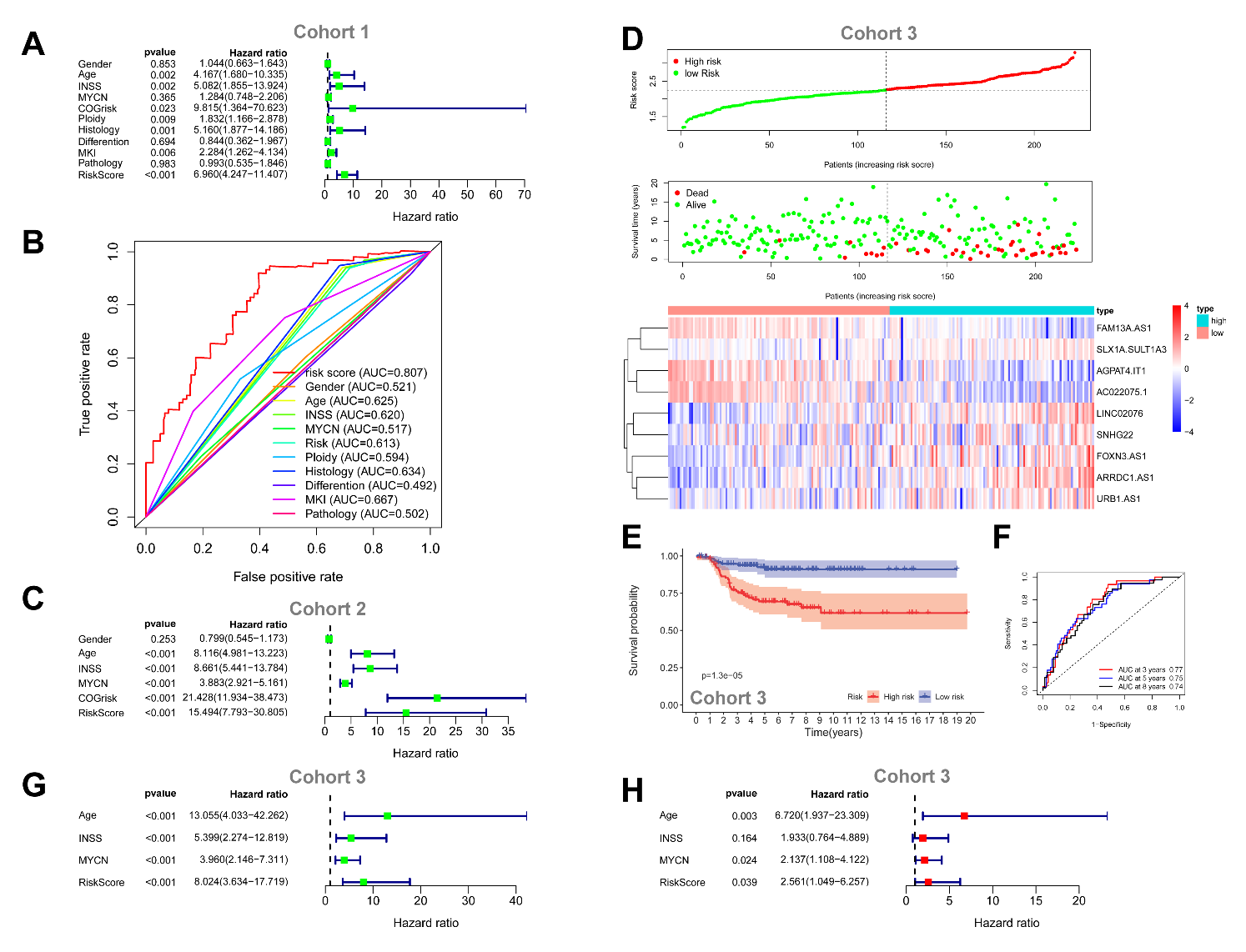
**

**Figure S5.** The prognostic value of the metabolism-related lncRNA signature. **(A)** The univariate Cox regression survival analysis in cohort 1. **(B)** The ROC curves for the metabolism-related lncRNA and other clinical risk factors in cohort 1. **(C)** The univariate Cox regression survival analysis in cohort 2. **(D)** The risk scores distribution, survival status of patients, and heatmap of genes expression pattern in cohort 3. **(E)** Kaplan-Meier plot for OS of patients in different risk groups of cohort 3. **(F)** Time-dependent ROC curves for the prognostic value of the metabolism-related lncRNA in cohort 3. **(G)** The univariate Cox regression survival analysis in cohort 3. **(H)** The multivariate Cox regression survival analysis in cohort 3.


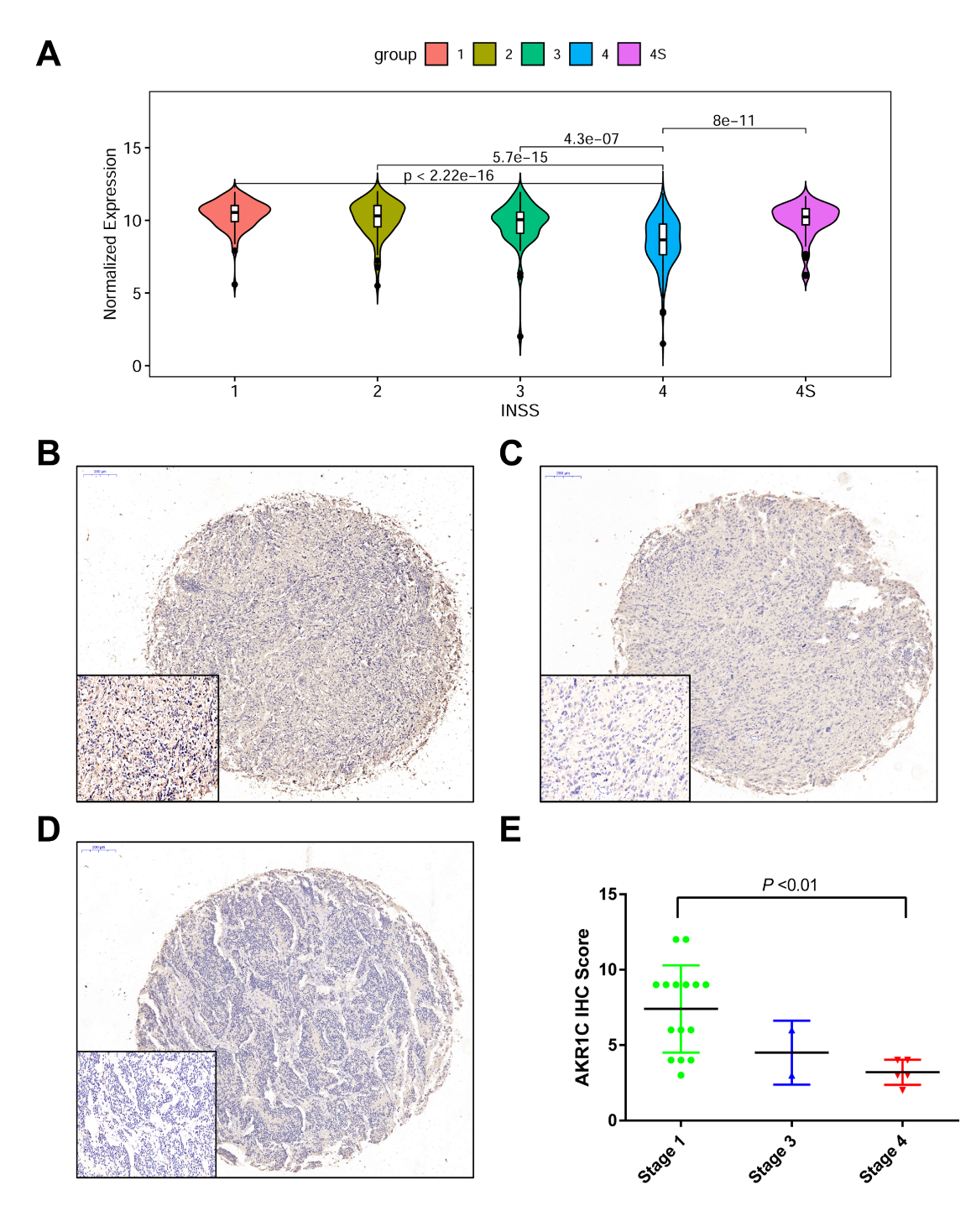


**Figure S6.** The IHC analysis of AKR1C1 expression in human NB tissues. Representative images of AKR1C1 staining are shown at × 100 magnification and insets are enlarged local images at × 400 magnification. **(A)** The relative expression levels of AKR1C1 in the largest microarray datasets (cohort 2). **(B)** The IHC of AKR1C1 expression in stage 1 NB tissues. **(C)** The IHC of AKR1C1 expression in stage 3 NB tissues. **(D)** The IHC of AKR1C1 expression in stage 4 NB tissues. **(E)** The comparison of the IHC scores of AKR1C1 expression between different stages NB tissues.

## Supplementary Table

**Table S1.** The clinical characteristics of the three cohorts.

|  | **Case No. of cohort 1 (%)** | **Case No. of cohort 2 (%)** | **Case No. of cohort 3 (%)** |
| --- | --- | --- | --- |
| **Age** |  |  |  |
| < 18 m | 29 (19.0%) | 300 (60.2%) | 103 (46.2%) |
| ≥ 18 m | 124 (81.0%) | 198 (39.8%) | 120 (53.8%) |
| **Gender** |  |  |  |
| Male | 89 (58.2%) | 287 (57.6%) | - |
| Female | 64 (41.8%) | 211 (42.4%) | - |
| **MYCN status** |  |  |  |
| Non-amplified | 121 (79.1%) | 401 (80.5%) | 176 (78.9%) |
| Amplified | 31 (20.3%) | 92 (18.5%) | 46 (20.6%) |
| **Risk** |  |  |  |
| Low | 27 (17.6%) | 322 (64.7%) | - |
| High | 126 (82.4%) | 176 (35.3%) | - |
| **INSS Stage** |  |  |  |
| 1 | 0 | 121 (24.3%) | 29 (13.0%) |
| 2 | 1 (0.7%) | 78 (15.7%) | 39 (17.5%) |
| 3 | 6 (3.9%) | 63 (12.7%) | 36 (16.1%) |
| 4 | 125 (81.7%) | 183 (36.7%) | 89 (39.9%) |
| 4S | 21 (13.7%) | 53 (10.6%) | 30 (13.5%) |
| **Vital status** |  |  |  |
| Dead | 77 (50.3%) | 105 (21.1%) | 42 (18.8%) |
| Alive | 76 (49.7%) | 393 (78.9%) | 181 (81.2%) |

**Table S2.** The characteristics of the 12 metabolism-related genes in the prognostic signature

| **Gene Symbol** | **Official Full Name** | **Ensembl id** | **Chromosomal Location** | **Coefficient** | **Univariate Cox survival analysis** | | |
| --- | --- | --- | --- | --- | --- | --- | --- |
|  |  |  |  |  | **HR** | **95% CI** | **p-value** |
| AKR1C1 | aldo-keto reductase family 1 member C1 | ENSG00000187134 | 10p15.1 | -0.1016 | 0.65 | 0.52-0.80 | < 0.001 |
| CDO1 | cysteine dioxygenase type 1 | ENSG00000129596 | 5q22.3 | -0.0475 | 0.78 | 0.67-0.91 | 0.002 |
| POLR2A | RNA polymerase II subunit A | ENSG00000181222 | 17p13.1 | -0.0227 | 0.93 | 0.88-0.98 | 0.012 |
| PGM2L1 | phosphoglucomutase 2 like 1 | ENSG00000165434 | 11q13.4 | -0.0152 | 0.84 | 0.76-0.93 | 0.001 |
| POLR2L | RNA polymerase II, I and III subunit L | ENSG00000177700 | 11p15.5 | 0.0013 | 1.04 | 1.02-1.07 | 0.001 |
| NME2 | NME/NM23 nucleoside diphosphate kinase 2 | ENSG00000243678 | 17q21.33 | 0.0029 | 1.04 | 1.03-1.06 | < 0.001 |
| PLCB3 | phospholipase C beta 3 | ENSG00000149782 | 11q13.1 | 0.0077 | 1.07 | 1.01-1.14 | 0.018 |
| PHPT1 | phosphohistidine phosphatase 1 | ENSG00000054148 | 9q34.3 | 0.0151 | 1.05 | 1.03-1.07 | < 0.001 |
| POLR2I | RNA polymerase II subunit I | ENSG00000105258 | 19q13.12 | 0.0192 | 1.15 | 1.09-1.21 | < 0.001 |
| MIF | macrophage migration inhibitory factor | ENSG00000240972 | 22q11.23 | 0.0192 | 1.05 | 1.03-1.07 | < 0.001 |
| LAP3 | leucine aminopeptidase 3 | ENSG00000002549 | 4p15.32 | 0.0305 | 1.08 | 1.02-1.15 | 0.008 |
| FADS2 | fatty acid desaturase 2 | ENSG00000134824 | 11q12.2 | 0.0354 | 1.09 | 1.04-1.13 | < 0.001 |

**Table S3.** The top 30 KEGG pathways enriched in high risk group

|  | **KEGG pathways enriched in high risk group** | **NES** | **p-value** | **FDR q-value** |
| --- | --- | --- | --- | --- |
| 1 | KEGG_OXIDATIVE_PHOSPHORYLATION | 2.62 | 0 | 0.002 |
| 2 | KEGG_SPLICEOSOME | 2.46 | 0.002 | 0.001 |
| 3 | KEGG_HUNTINGTONS_DISEASE | 2.37 | 0 | 0.001 |
| 4 | KEGG_RNA_POLYMERASE | 2.34 | 0 | 0 |
| 5 | KEGG_CELL_CYCLE | 2.22 | 0 | 0.002 |
| 6 | KEGG_RNA_DEGRADATION | 2.2 | 0.002 | 0.002 |
| 7 | KEGG_PARKINSONS_DISEASE | 2.15 | 0.004 | 0.003 |
| 8 | KEGG_NUCLEOTIDE_EXCISION_REPAIR | 2.11 | 0.002 | 0.003 |
| 9 | KEGG_HOMOLOGOUS_RECOMBINATION | 2.06 | 0.004 | 0.006 |
| 10 | KEGG_BASE_EXCISION_REPAIR | 2.06 | 0 | 0.005 |
| 11 | KEGG_AMINOACYL_TRNA_BIOSYNTHESIS | 2.05 | 0.004 | 0.006 |
| 12 | KEGG_DNA_REPLICATION | 2.02 | 0.008 | 0.007 |
| 13 | KEGG_PYRIMIDINE_METABOLISM | 1.99 | 0 | 0.008 |
| 14 | KEGG_LYSINE_DEGRADATION | 1.93 | 0.009 | 0.012 |
| 15 | KEGG_PROTEASOME | 1.88 | 0.023 | 0.015 |
| 16 | KEGG_MISMATCH_REPAIR | 1.84 | 0.018 | 0.02 |
| 17 | KEGG_ONE_CARBON_POOL_BY_FOLATE | 1.83 | 0.013 | 0.021 |
| 18 | KEGG_CITRATE_CYCLE_TCA_CYCLE | 1.83 | 0.014 | 0.02 |
| 19 | KEGG_PROTEIN_EXPORT | 1.79 | 0.014 | 0.026 |
| 20 | KEGG_STEROID_BIOSYNTHESIS | 1.74 | 0.02 | 0.031 |
| 21 | KEGG_P53_SIGNALING_PATHWAY | 1.73 | 0.002 | 0.03 |
| 22 | KEGG_BASAL_TRANSCRIPTION_FACTORS | 1.72 | 0.006 | 0.03 |
| 23 | KEGG_SELENOAMINO_ACID_METABOLISM | 1.69 | 0.014 | 0.035 |
| 24 | KEGG_GLYCOSYLPHOSPHATIDYLINOSITOL_GPI_ANCHOR_BIOSYNTHESIS | 1.6 | 0.02 | 0.055 |
| 25 | KEGG_UBIQUITIN_MEDIATED_PROTEOLYSIS | 1.59 | 0.023 | 0.056 |
| 26 | KEGG_PROGESTERONE_MEDIATED_OOCYTE_MATURATION | 1.59 | 0.031 | 0.055 |
| 27 | KEGG_N_GLYCAN_BIOSYNTHESIS | 1.59 | 0.038 | 0.054 |
| 28 | KEGG_ALZHEIMERS_DISEASE | 1.59 | 0.015 | 0.052 |
| 29 | KEGG_RIBOSOME | 1.58 | 0.078 | 0.054 |
| 30 | KEGG_OOCYTE_MEIOSIS | 1.56 | 0.014 | 0.056 |

**Table S4.** The top 30 KEGG pathways enriched in low risk group

|  | **KEGG pathways enriched in low risk group** | **NES** | **p-value** | **FDR q-value** |
| --- | --- | --- | --- | --- |
| 1 | KEGG_ALDOSTERONE_REGULATED_SODIUM_REABSORPTION | -1.51 | 0.01 | 1 |
| 2 | KEGG_CELL_ADHESION_MOLECULES_CAMS | -1.47 | 0.032 | 1 |
| 3 | KEGG_ECM_RECEPTOR_INTERACTION | -1.43 | 0.06 | 1 |
| 4 | KEGG_ARRHYTHMOGENIC_RIGHT_VENTRICULAR_CARDIOMYOPATHY_ARVC | -1.41 | 0.034 | 1 |
| 5 | KEGG_PHOSPHATIDYLINOSITOL_SIGNALING_SYSTEM | -1.41 | 0.051 | 1 |
| 6 | KEGG_GRAFT_VERSUS_HOST_DISEASE | -1.41 | 0.105 | 1 |
| 7 | KEGG_ABC_TRANSPORTERS | -1.38 | 0.088 | 1 |
| 8 | KEGG_ASTHMA | -1.34 | 0.087 | 1 |
| 9 | KEGG_HEMATOPOIETIC_CELL_LINEAGE | -1.34 | 0.128 | 1 |
| 10 | KEGG_AXON_GUIDANCE | -1.33 | 0.079 | 1 |
| 11 | KEGG_TYPE_I_DIABETES_MELLITUS | -1.33 | 0.14 | 1 |
| 12 | KEGG_LEUKOCYTE_TRANSENDOTHELIAL_MIGRATION | -1.32 | 0.093 | 1 |
| 13 | KEGG_ADHERENS_JUNCTION | -1.32 | 0.132 | 0.975 |
| 14 | KEGG_INTESTINAL_IMMUNE_NETWORK_FOR_IGA_PRODUCTION | -1.31 | 0.167 | 0.94 |
| 15 | KEGG_COMPLEMENT_AND_COAGULATION_CASCADES | -1.29 | 0.175 | 0.983 |
| 16 | KEGG_VIRAL_MYOCARDITIS | -1.29 | 0.159 | 0.927 |
| 17 | KEGG_DILATED_CARDIOMYOPATHY | -1.29 | 0.102 | 0.874 |
| 18 | KEGG_RENIN_ANGIOTENSIN_SYSTEM | -1.28 | 0.145 | 0.865 |
| 19 | KEGG_AUTOIMMUNE_THYROID_DISEASE | -1.28 | 0.14 | 0.827 |
| 20 | KEGG_T_CELL_RECEPTOR_SIGNALING_PATHWAY | -1.28 | 0.216 | 0.79 |
| 21 | KEGG_HYPERTROPHIC_CARDIOMYOPATHY_HCM | -1.27 | 0.118 | 0.761 |
| 22 | KEGG_FOCAL_ADHESION | -1.27 | 0.169 | 0.747 |
| 23 | KEGG_THYROID_CANCER | -1.25 | 0.179 | 0.767 |
| 24 | KEGG_ALLOGRAFT_REJECTION | -1.24 | 0.22 | 0.783 |
| 25 | KEGG_PANTOTHENATE_AND_COA_BIOSYNTHESIS | -1.2 | 0.234 | 0.883 |
| 26 | KEGG_DORSO_VENTRAL_AXIS_FORMATION | -1.2 | 0.238 | 0.863 |
| 27 | KEGG_LONG_TERM_POTENTIATION | -1.19 | 0.216 | 0.871 |
| 28 | KEGG_FC_GAMMA_R_MEDIATED_PHAGOCYTOSIS | -1.18 | 0.276 | 0.88 |
| 29 | KEGG_NICOTINATE_AND_NICOTINAMIDE_METABOLISM | -1.17 | 0.285 | 0.89 |
| 30 | KEGG_NOTCH_SIGNALING_PATHWAY | -1.17 | 0.295 | 0.863 |

**Table S5.** The KEGG pathways enriched in high risk group by ssGSEA analysis

| **KEGG ID** | **KEGG pathways description** | **NES** | **p-value** | **q-value** |
| --- | --- | --- | --- | --- |
| hsa03030 | DNA replication | 2.69 | 1.00E-10 | 3.40E-09 |
| hsa03430 | Mismatch repair | 2.09 | 2.31E-04 | 1.31E-03 |
| hsa03410 | Base excision repair | 2.25 | 8.72E-06 | 8.48E-05 |
| hsa03008 | Ribosome biogenesis in eukaryotes | 2.62 | 1.00E-10 | 3.40E-09 |
| hsa03020 | RNA polymerase | 2.15 | 5.85E-05 | 4.01E-04 |
| hsa00670 | One carbon pool by folate | 1.88 | 1.31E-03 | 4.24E-03 |
| hsa03440 | Homologous recombination | 2.27 | 9.86E-07 | 1.34E-05 |
| hsa04110 | Cell cycle | 2.77 | 1.00E-10 | 3.40E-09 |
| hsa00970 | Aminoacyl-tRNA biosynthesis | 1.99 | 4.24E-04 | 1.84E-03 |
| hsa03010 | Ribosome | 2.79 | 1.00E-10 | 3.40E-09 |
| hsa03460 | Fanconi anemia pathway | 2.35 | 1.27E-07 | 1.86E-06 |
| hsa00100 | Steroid biosynthesis | 1.87 | 1.51E-03 | 4.76E-03 |
| hsa03420 | Nucleotide excision repair | 2.23 | 2.44E-06 | 2.63E-05 |
| hsa03013 | RNA transport | 2.56 | 1.00E-10 | 3.40E-09 |
| hsa03040 | Spliceosome | 2.46 | 1.00E-10 | 3.40E-09 |
| hsa00190 | Oxidative phosphorylation | 2.33 | 7.67E-10 | 1.96E-08 |
| hsa03050 | Proteasome | 1.85 | 1.33E-03 | 4.24E-03 |
| hsa04115 | p53 signaling pathway | 1.95 | 2.57E-05 | 2.16E-04 |
| hsa03018 | RNA degradation | 1.94 | 4.25E-05 | 3.10E-04 |
| hsa03015 | mRNA surveillance pathway | 1.81 | 1.81E-04 | 1.09E-03 |
| hsa04914 | Progesterone-mediated oocyte maturation | 1.80 | 3.16E-04 | 1.59E-03 |
| hsa04114 | Oocyte meiosis | 1.71 | 5.15E-04 | 2.06E-03 |
| hsa04218 | Cellular senescence | 1.71 | 5.89E-05 | 4.01E-04 |
| hsa04141 | Protein processing in endoplasmic reticulum | 1.67 | 3.30E-04 | 1.60E-03 |
| hsa04714 | Thermogenesis | 1.74 | 1.24E-05 | 1.15E-04 |
| hsa04120 | Ubiquitin mediated proteolysis | 1.61 | 7.35E-04 | 2.78E-03 |
| hsa05016 | Huntington disease | 1.70 | 1.92E-06 | 2.25E-05 |
| hsa05012 | Parkinson disease | 1.58 | 2.20E-04 | 1.28E-03 |

**Table S6.** The top 30 KEGG pathways enriched in low risk group by ssGSEA analysis

| **KEGG ID** | **KEGG pathways description** | **NES** | **p-value** | **q-value** |
| --- | --- | --- | --- | --- |
| hsa05310 | Asthma | -2.09 | 3.12E-05 | 2.45E-04 |
| hsa05332 | Graft-versus-host disease | -2.04 | 1.74E-04 | 1.08E-03 |
| hsa05150 | Staphylococcus aureus infection | -2.36 | 1.03E-09 | 2.33E-08 |
| hsa04610 | Complement and coagulation cascades | -2.29 | 6.23E-09 | 1.19E-07 |
| hsa04640 | Hematopoietic cell lineage | -2.34 | 6.32E-10 | 1.84E-08 |
| hsa05321 | Inflammatory bowel disease (IBD) | -2.11 | 1.89E-06 | 2.25E-05 |
| hsa04960 | Aldosterone-regulated sodium reabsorption | -1.91 | 5.14E-04 | 2.06E-03 |
| hsa04672 | Intestinal immune network for IgA production | -1.96 | 1.36E-04 | 8.97E-04 |
| hsa04979 | Cholesterol metabolism | -1.99 | 1.98E-05 | 1.76E-04 |
| hsa04512 | ECM-receptor interaction | -2.19 | 9.16E-08 | 1.44E-06 |
| hsa00982 | Drug metabolism - cytochrome P450 | -1.98 | 3.83E-05 | 2.90E-04 |
| hsa05320 | Autoimmune thyroid disease | -1.87 | 3.94E-04 | 1.79E-03 |
| hsa05322 | Systemic lupus erythematosus | -1.85 | 6.59E-04 | 2.59E-03 |
| hsa04514 | Cell adhesion molecules (CAMs) | -2.14 | 6.41E-09 | 1.19E-07 |
| hsa04750 | Inflammatory mediator regulation of TRP channels | -2.03 | 4.81E-06 | 4.91E-05 |
| hsa04659 | Th17 cell differentiation | -2.03 | 1.98E-06 | 2.25E-05 |
| hsa05204 | Chemical carcinogenesis | -1.84 | 1.56E-04 | 9.94E-04 |
| hsa04658 | Th1 and Th2 cell differentiation | -1.91 | 2.64E-05 | 2.16E-04 |
| hsa05031 | Amphetamine addiction | -1.81 | 3.84E-04 | 1.78E-03 |
| hsa00980 | Metabolism of xenobiotics by cytochrome P450 | -1.74 | 1.23E-03 | 4.10E-03 |
| hsa05412 | Arrhythmogenic right ventricular cardiomyopathy (ARVC) | -1.75 | 1.56E-03 | 4.76E-03 |
| hsa05146 | Amoebiasis | -1.80 | 2.80E-04 | 1.49E-03 |
| hsa04974 | Protein digestion and absorption | -1.74 | 3.77E-04 | 1.78E-03 |
| hsa04925 | Aldosterone synthesis and secretion | -1.72 | 1.13E-03 | 3.98E-03 |
| hsa04650 | Natural killer cell mediated cytotoxicity | -1.76 | 5.10E-04 | 2.06E-03 |
| hsa05410 | Hypertrophic cardiomyopathy (HCM) | -1.70 | 9.36E-04 | 3.48E-03 |
| hsa04060 | Cytokine-cytokine receptor interaction | -1.92 | 7.19E-09 | 1.22E-07 |
| hsa04668 | TNF signaling pathway | -1.73 | 4.20E-04 | 1.84E-03 |
| hsa04660 | T cell receptor signaling pathway | -1.70 | 1.03E-03 | 3.68E-03 |
| hsa04061 | Viral protein interaction with cytokine and cytokine receptor | -1.69 | 1.60E-03 | 4.81E-03 |

**Table S7.** The top 30 GO biological processes enriched in high risk group by ssGSEA analysis

| **GO ID** | **GO biological process description** | **NES** | **p-value** | **q-value** |
| --- | --- | --- | --- | --- |
| GO:0051383 | kinetochore organization | 2.60 | 2.72E-10 | 9.64E-09 |
| GO:0000727 | double-strand break repair via break-induced replication | 2.16 | 2.25E-05 | 2.52E-04 |
| GO:2000105 | positive regulation of DNA-dependent DNA replication | 2.15 | 2.36E-05 | 2.62E-04 |
| GO:0051382 | kinetochore assembly | 2.27 | 1.70E-06 | 2.57E-05 |
| GO:0000076 | DNA replication checkpoint | 2.28 | 2.32E-06 | 3.38E-05 |
| GO:0034427 | nuclear-transcribed mRNA catabolic process, exonucleolytic, 3'-5' | 2.01 | 2.80E-04 | 2.01E-03 |
| GO:0006271 | DNA strand elongation involved in DNA replication | 2.33 | 2.28E-06 | 3.35E-05 |
| GO:1902969 | mitotic DNA replication | 2.24 | 3.81E-06 | 5.22E-05 |
| GO:0031055 | chromatin remodeling at centromere | 2.67 | 2.53E-10 | 9.04E-09 |
| GO:0032201 | telomere maintenance via semi-conservative replication | 2.54 | 1.84E-09 | 5.21E-08 |
| GO:0034080 | CENP-A containing nucleosome assembly | 2.60 | 8.74E-10 | 2.75E-08 |
| GO:0061641 | CENP-A containing chromatin organization | 2.60 | 8.74E-10 | 2.75E-08 |
| GO:0045835 | negative regulation of meiotic nuclear division | 1.96 | 6.82E-04 | 4.07E-03 |
| GO:0009113 | purine nucleobase biosynthetic process | 2.00 | 5.49E-04 | 3.45E-03 |
| GO:0033314 | mitotic DNA replication checkpoint | 1.94 | 8.36E-04 | 4.75E-03 |
| GO:2000816 | negative regulation of mitotic sister chromatid separation | 2.76 | 1.00E-10 | 3.86E-09 |
| GO:1905819 | negative regulation of chromosome separation | 2.76 | 1.00E-10 | 3.86E-09 |
| GO:0070601 | centromeric sister chromatid cohesion | 1.98 | 6.71E-04 | 4.02E-03 |
| GO:0033048 | negative regulation of mitotic sister chromatid segregation | 2.80 | 1.00E-10 | 3.86E-09 |
| GO:0033046 | negative regulation of sister chromatid segregation | 2.83 | 1.00E-10 | 3.86E-09 |
| GO:0051985 | negative regulation of chromosome segregation | 2.84 | 1.00E-10 | 3.86E-09 |
| GO:0034508 | centromere complex assembly | 2.74 | 1.00E-10 | 3.86E-09 |
| GO:0090231 | regulation of spindle checkpoint | 2.09 | 8.40E-05 | 7.68E-04 |
| GO:0090266 | regulation of mitotic cell cycle spindle assembly checkpoint | 2.09 | 8.40E-05 | 7.68E-04 |
| GO:1903504 | regulation of mitotic spindle checkpoint | 2.09 | 8.40E-05 | 7.68E-04 |
| GO:0035358 | regulation of peroxisome proliferator activated receptor signaling pathway | 2.09 | 8.53E-05 | 7.76E-04 |
| GO:1904668 | positive regulation of ubiquitin protein ligase activity | 1.99 | 4.28E-04 | 2.84E-03 |
| GO:0043144 | snoRNA processing | 2.04 | 2.83E-04 | 2.02E-03 |
| GO:0006268 | DNA unwinding involved in DNA replication | 2.07 | 1.41E-04 | 1.17E-03 |
| GO:0034724 | DNA replication-independent nucleosome organization | 2.68 | 1.00E-10 | 3.86E-09 |

**Table S8.** The top 30 GO biological processes enriched in low risk group by ssGSEA analysis

| **GO ID** | **GO biological process description** | **NES** | **p-value** | **q-value** |
| --- | --- | --- | --- | --- |
| GO:0034370 | triglyceride-rich lipoprotein particle remodeling | -2.05 | 8.76E-05 | 7.90E-04 |
| GO:0034372 | very-low-density lipoprotein particle remodeling | -1.95 | 3.30E-04 | 2.30E-03 |
| GO:2000402 | negative regulation of lymphocyte migration | -2.01 | 2.45E-04 | 1.82E-03 |
| GO:0071073 | positive regulation of phospholipid biosynthetic process | -1.93 | 4.98E-04 | 3.19E-03 |
| GO:0060192 | negative regulation of lipase activity | -1.99 | 1.30E-04 | 1.09E-03 |
| GO:0051923 | sulfation | -1.94 | 6.92E-04 | 4.11E-03 |
| GO:0046629 | gamma-delta T cell activation | -1.85 | 1.32E-03 | 6.84E-03 |
| GO:0051004 | regulation of lipoprotein lipase activity | -2.06 | 3.18E-05 | 3.39E-04 |
| GO:0042228 | interleukin-8 biosynthetic process | -1.88 | 6.70E-04 | 4.02E-03 |
| GO:0006883 | cellular sodium ion homeostasis | -1.87 | 6.70E-04 | 4.02E-03 |
| GO:0045414 | regulation of interleukin-8 biosynthetic process | -1.87 | 8.37E-04 | 4.75E-03 |
| GO:0071071 | regulation of phospholipid biosynthetic process | -1.87 | 6.86E-04 | 4.09E-03 |
| GO:0031579 | membrane raft organization | -1.97 | 1.75E-04 | 1.38E-03 |
| GO:0034375 | high-density lipoprotein particle remodeling | -1.88 | 5.38E-04 | 3.41E-03 |
| GO:0042094 | interleukin-2 biosynthetic process | -1.99 | 2.27E-04 | 1.70E-03 |
| GO:0051482 | positive regulation of cytosolic calcium ion concentration involved in phospholipase C-activating G protein-coupled signaling pathway | -2.10 | 8.56E-06 | 1.09E-04 |
| GO:2000316 | regulation of T-helper 17 type immune response | -1.89 | 9.79E-04 | 5.43E-03 |
| GO:0045076 | regulation of interleukin-2 biosynthetic process | -1.87 | 1.14E-03 | 6.11E-03 |
| GO:0009713 | catechol-containing compound biosynthetic process | -1.90 | 9.94E-04 | 5.47E-03 |
| GO:0042423 | catecholamine biosynthetic process | -1.90 | 9.94E-04 | 5.47E-03 |
| GO:0035025 | positive regulation of Rho protein signal transduction | -2.03 | 5.69E-05 | 5.54E-04 |
| GO:0045061 | thymic T cell selection | -1.91 | 4.71E-04 | 3.04E-03 |
| GO:0050951 | sensory perception of temperature stimulus | -1.90 | 5.89E-04 | 3.64E-03 |
| GO:0086010 | membrane depolarization during action potential | -2.08 | 1.05E-05 | 1.31E-04 |
| GO:0086012 | membrane depolarization during cardiac muscle cell action potential | -1.87 | 8.99E-04 | 5.04E-03 |
| GO:0002719 | negative regulation of cytokine production involved in immune response | -1.91 | 3.73E-04 | 2.53E-03 |
| GO:0055090 | acylglycerol homeostasis | -2.04 | 2.40E-05 | 2.65E-04 |
| GO:0070328 | triglyceride homeostasis | -2.04 | 2.40E-05 | 2.65E-04 |
| GO:0016339 | calcium-dependent cell-cell adhesion via plasma membrane cell adhesion molecules | -2.16 | 1.51E-06 | 2.29E-05 |
| GO:0002686 | negative regulation of leukocyte migration | -2.14 | 6.56E-06 | 8.57E-05 |

**Table S9.** The characteristics of the 9 metabolism-related lncRNAs in the prognostic signature

| **Gene Symbol** | **Official Full Name** | **Ensembl id** | **Chromosomal Location** | **Coefficient** | **Univariate Cox survival analysis** | | |
| --- | --- | --- | --- | --- | --- | --- | --- |
|  |  |  |  |  | **HR** | **95% CI** | **p-value** |
| FAM13A-AS1 | FAM13A antisense RNA 1 | ENSG00000248019 | 4q22.1 | -0.0932 | 0.67 | 0.54-0.84 | 0.001 |
| AGPAT4-IT1 | AGPAT4 intronic transcript 1 | ENSG00000279355 | 6q26 | -0.0650 | 0.60 | 0.43-0.83 | 0.002 |
| KLRK1-AS1 (AC022075.1) | KLRK1 antisense RNA 1 | ENSG00000245648 | 12p13.2 | -0.0600 | 0.65 | 0.51-0.84 | 0.001 |
| URB1-AS1 | URB1 antisense RNA 1 | ENSG00000256073 | 21q22.11 | 0.0196 | 1.26 | 1.11-1.43 | < 0.001 |
| FOXN3-AS1 | FOXN3 antisense RNA 1 | ENSG00000258920 | 14q32.11 | 0.0386 | 1.53 | 1.21-1.94 | < 0.001 |
| SNHG22 | small nucleolar RNA host gene 22 | ENSG00000267322 | 18q21.1 | 0.0577 | 1.32 | 1.16-1.50 | < 0.001 |
| LINC02076 | long intergenic non-protein coding RNA 2076 | ENSG00000220161 | 17p11.2 | 0.1056 | 1.43 | 1.24-1.64 | < 0.001 |
| SLX1A-SULT1A3 | SLX1A-SULT1A3 readthrough | ENSG00000213599 | 16p11.2 | 0.1167 | 1.73 | 1.34-2.22 | < 0.001 |
| ARRDC1-AS1 | ARRDC1 antisense RNA 1 | ENSG00000203993 | 9q34.3 | 0.1185 | 1.48 | 1.22-1.80 | < 0.001 |
